# Supplementary material for: Computational framework for prioritizing candidate compounds overcoming the resistance of pancancer immunotherapy
Source: Cell Rep Med. 2025 Aug 5;6(8):102276. doi: 10.1016/j.xcrm.2025.102276 (PMC12432370; doi:10.1016/j.xcrm.2025.102276)
Supplement: Document S1. Figures S1–S7 and Tables S2, S5, S8, and S9 [file mmc1.pdf]

Cell Reports Medicine, Volume *6*

Supplemental information

Computational framework for prioritizing candidate compounds overcoming the resistance of pancancer immunotherapy

Fangyoumin Feng, Tian He, Ping Lin, Jinwu Hu, Bihan Shen, Zhixuan Tang, Jian Zhou, Jia Fan, Bo Hu, and Hong Li

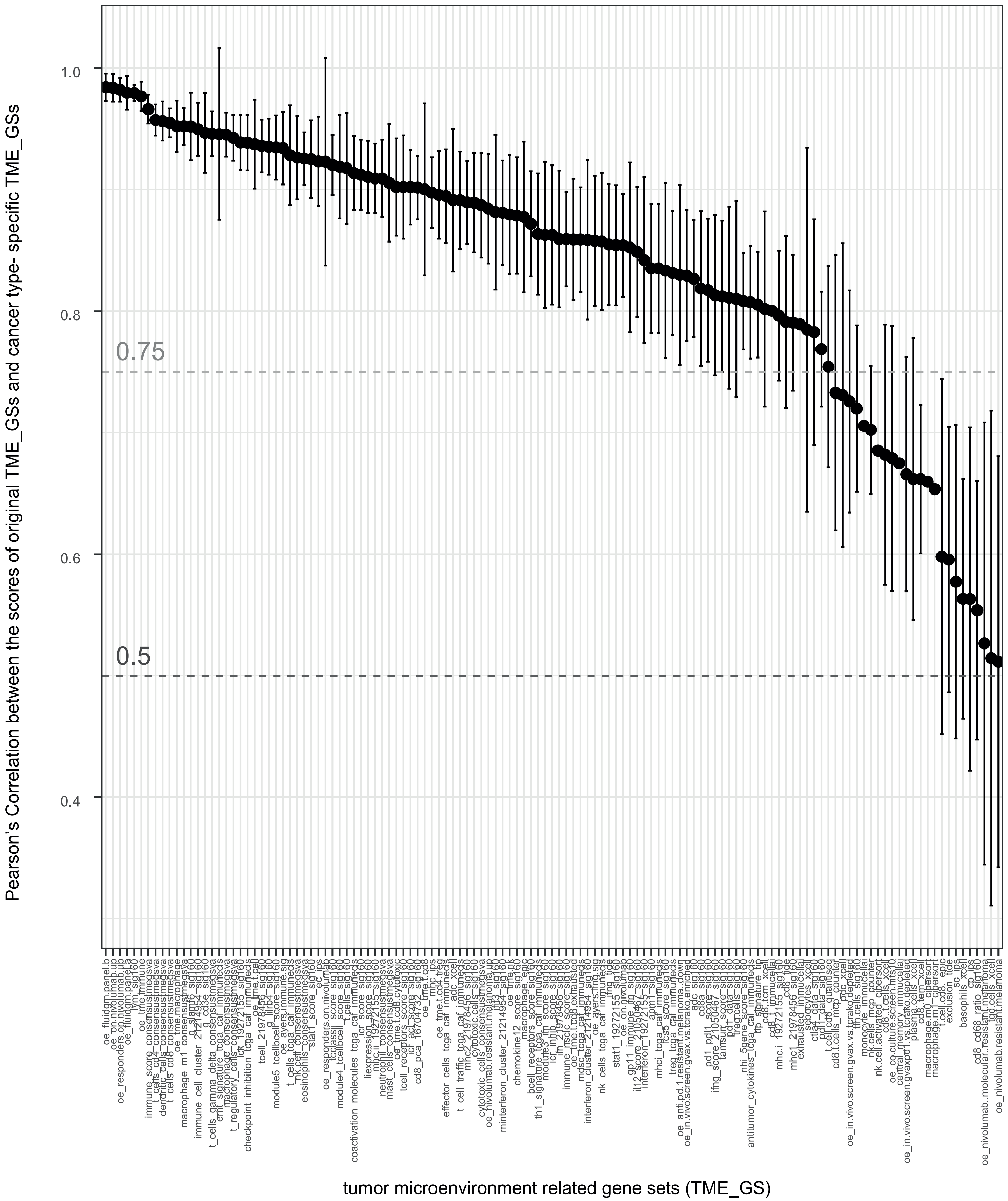

Figure S1. Pearson correlation coefficient (PCC) between the scores of the original signatures and the scores of the extended cancer type-specific signatures.Related to Figure 1. The data bar indicates the range of PCCs among multiple cancer types.

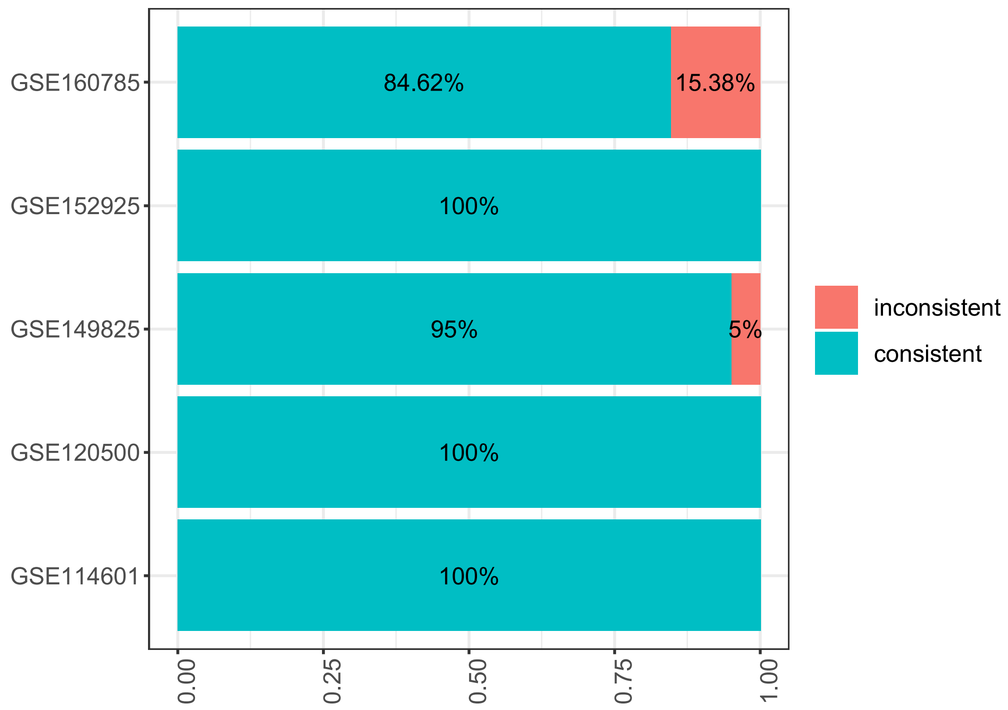

Figure S2. Consistency of drug-induced IGeS expression changes between mice and cell lines. Related to Figure 3.

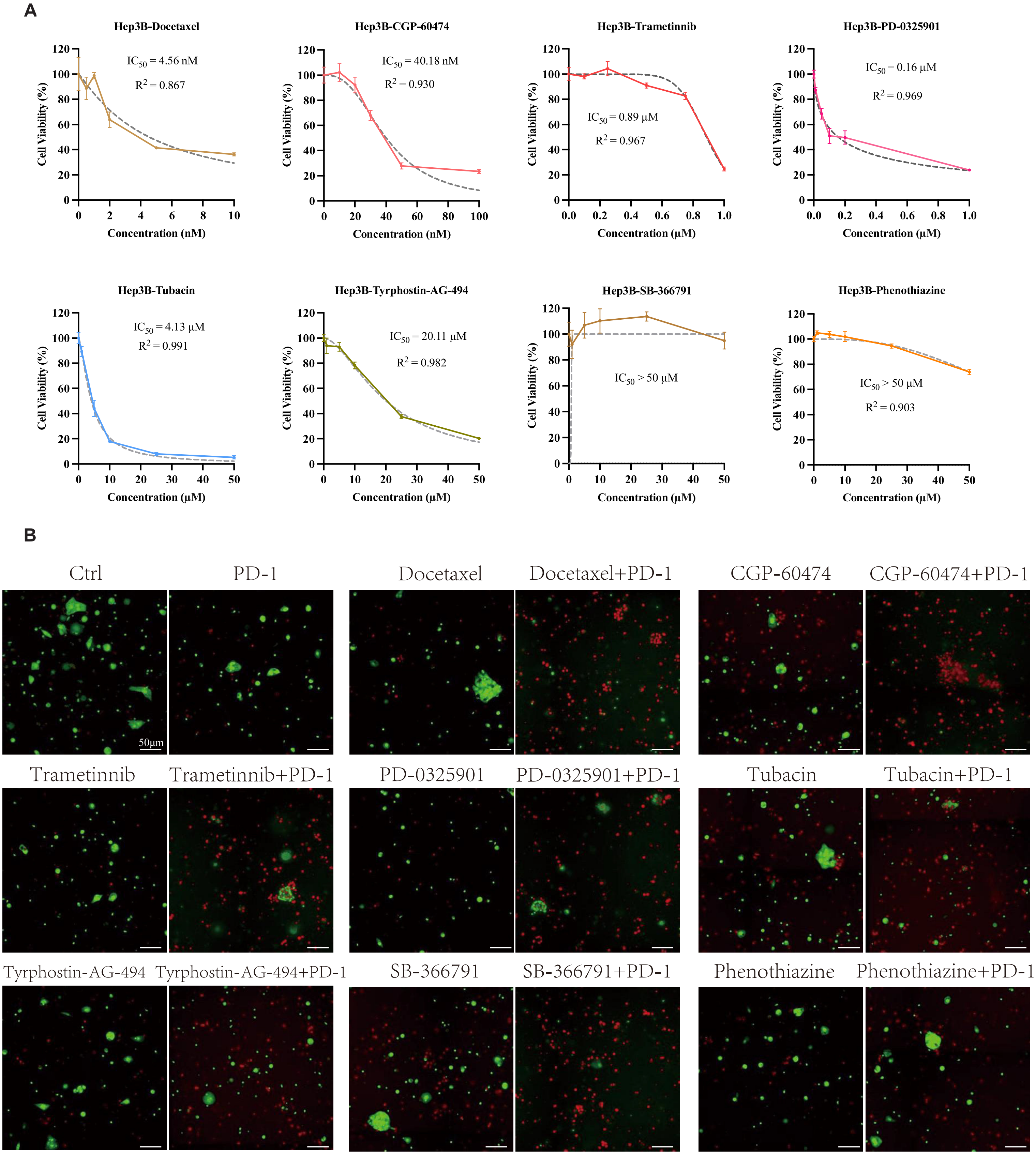

Figure S3. Drug test results in cell lines and PDOTs. Related to Figure 5. (A), The IC50 values of the selected compounds in the Hep3B cell line. **(B),** Representative pictures of PDOTs live/dead analysis via high-content screening. Live cells were stained green (AO), while dead cells were stained red (PI). Data are represented as mean ± SD. Scale bar: 50µm.

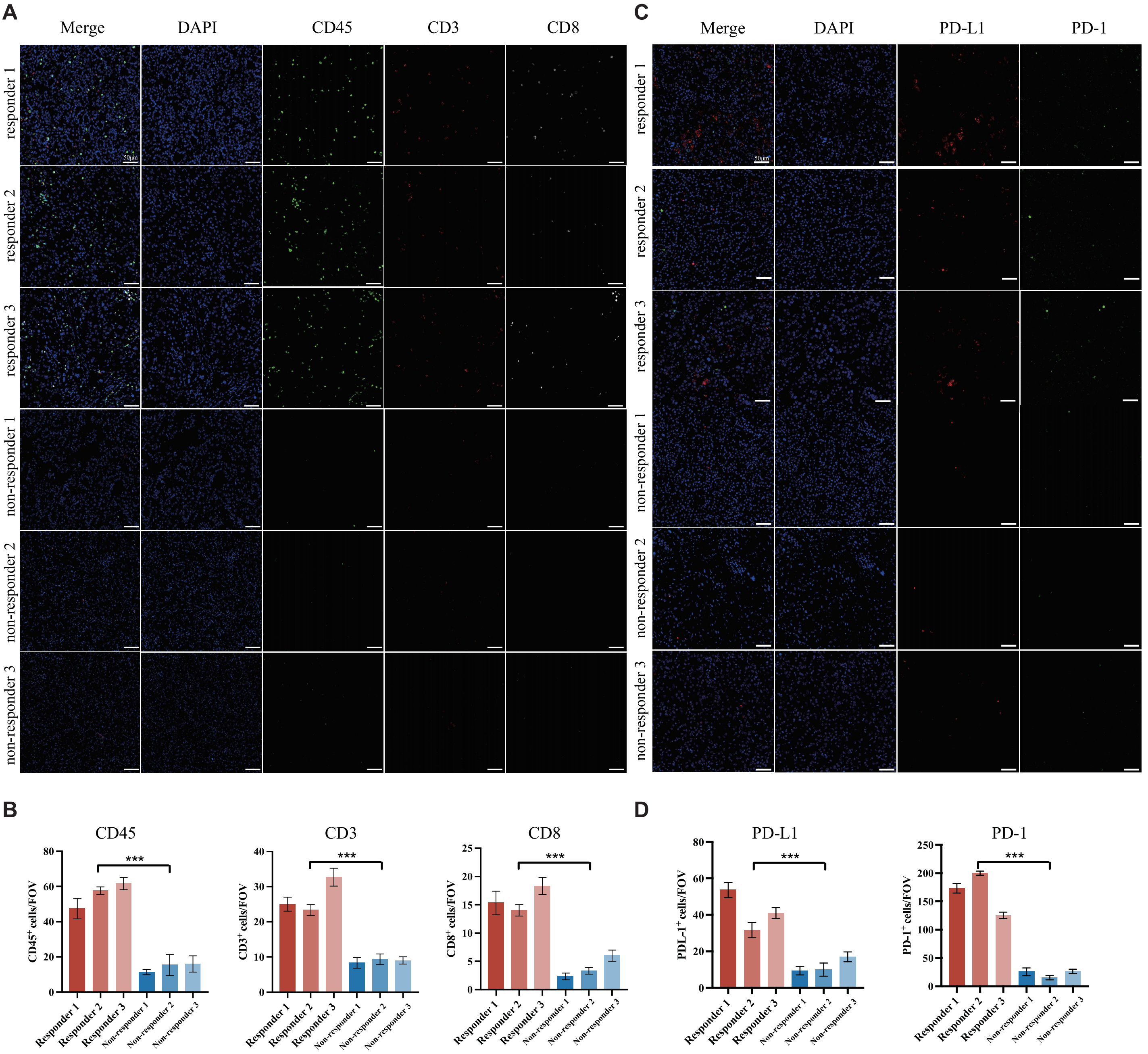

Figure S4. Comparison of immune infiltration between three anti-PD-1-resistant patients and three responding patients. Related to Figure 5. (A), Representative images of CD45, CD3 and CD8 immunofluorescence staining. **(B),** Statistical comparison of PD-1/PD-L1 expression. **(C),** Representative images of PD-1/PD-L1 immunofluorescence staining. **(D),** Statistical comparison of CD45, CD3 and CD8 expression. Scale bar: 50 μm. Data are represented as mean ± SD. Asterisks indicate the level of statistical significance: *** $p < 0.001$.

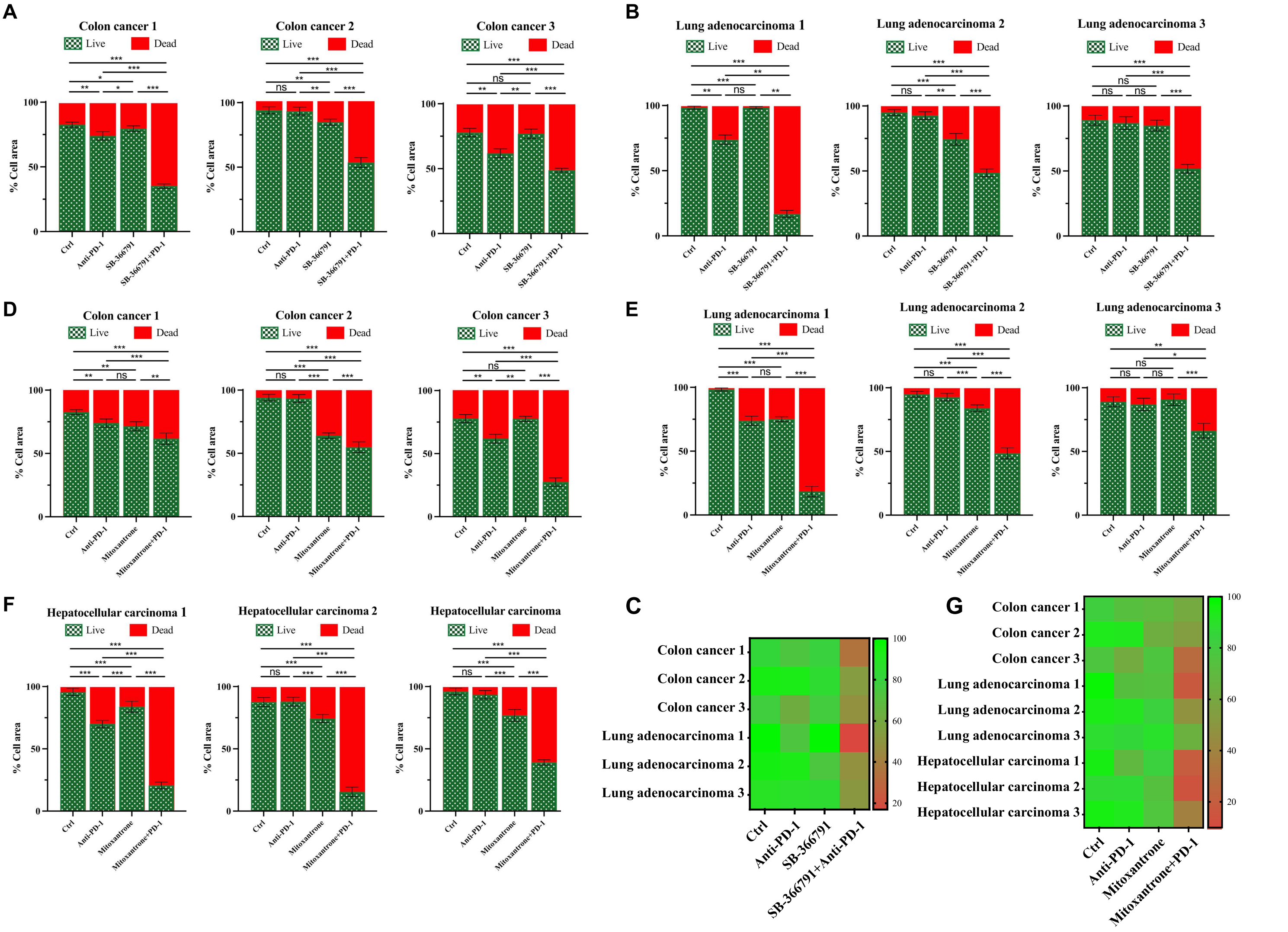

Figure S5. Validation of the effects of SB-366791 and mitoxantrone by PDOTs. Related to Figure 5. (A)-(B), PDOTs test results of SB-366791 based on colon cancer and lung adenocarcinoma samples. **(C),** Heatmap of SB-366791 PDOTs test results. **(D)–(F),** PDOTs test results of mitoxantrone in colon cancer, lung adenocarcinoma and hepatocellular carcinoma. **(G),** Heatmap of the mitoxantrone PDOTs test results. Data are represented as mean ± SD. Asterisks indicate the level of statistical significance: * $p < 0.05$, ** $p < 0.01$, *** $p < 0.001$, **** $p < 0.0001$, ns, not significant.

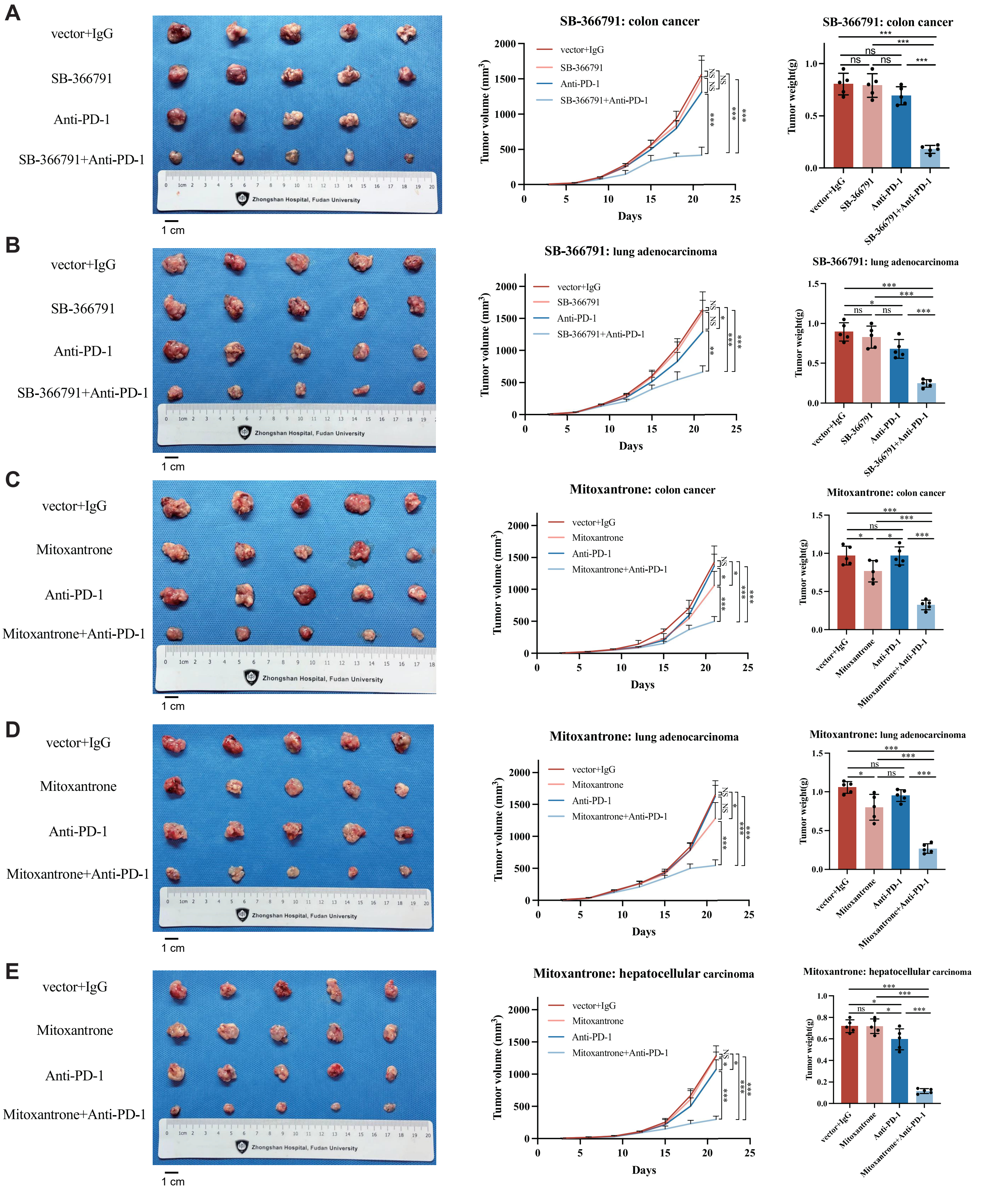

Figure S6. Validation of the effects of SB-366791 and mitoxantrone in a subcutaneous mouse model in four treatment groups: control, anti-PD-1, the candidate compound, and the combination of candidate and anti-PD-1.Related to Figure 6. (A)-(B), Results for SB-366791 in colon cancer and lung adenocarcinoma models. **(C)-(E),** Results for mitoxantrone in colon cancer, lung adenocarcinoma and hepatocellular carcinoma models. Data are represented as mean ± SD. Asterisks indicate the level of statistical significance: * $p < 0.05$, ** $p < 0.01$, *** $p < 0.001$, **** $p < 0.0001$, ns, not significant. Scale bar: 1 cm.

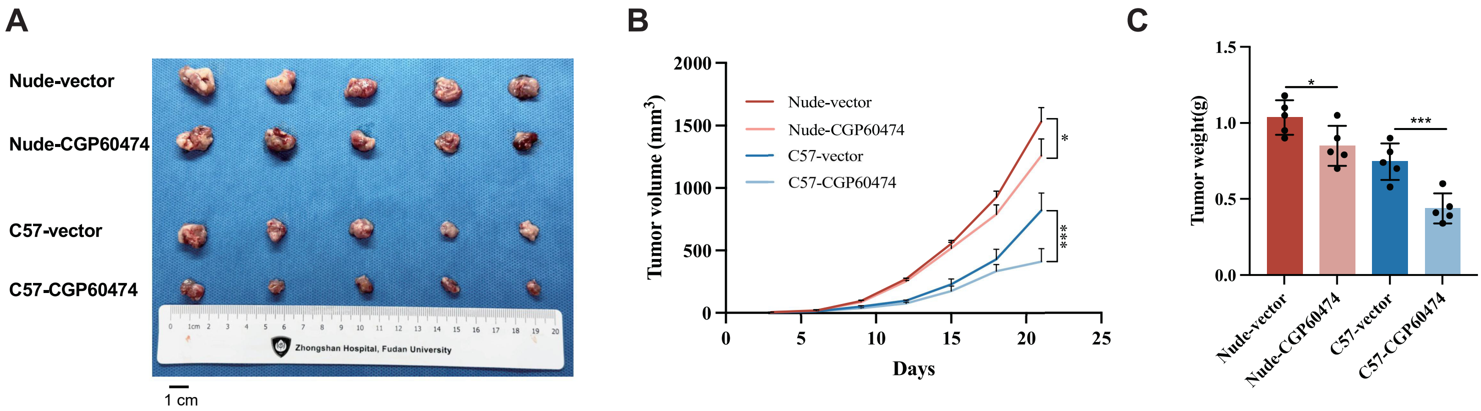

Figure S7. Representative images and quantification of subcutaneous tumors established in BALB/c nude and C57BL/6J mice. Related to Figure 6. (A), Tumour weights before and after CGP-60474 treatment. **(B),** Growth curves of the mice. **(C),** Statistical comparison of tumor weights among the different groups. To establish tumors, BALB/c nude and C57BL/6J mouse were injected subcutaneously in the right underarm with 1 × 106 Hepa1-6 cells in RPMI 1640 serum-free medium (200μL). Two weeks after injection, mice bearing subcutaneous tumors were randomized into groups. Treatments were administered CGP-60474(10 mg/kg) intraperitoneally every two days and seven times in total. Data are represented as mean ± SD. Asterisks indicate the level of statistical significance: * $p < 0.05$, ** $p < 0.01$, *** $p < 0.001$, **** $p < 0.0001$, ns, not significant. Scale bar: 1 cm.

Table S2. Manually collected immunotherapy datasets with known treatment effects and expression profiles. Related to Figures 1 and 2.

cohorts	dataset	source_PMID	cancer_type	treatment_type	responders	non-responders
discovery cohorts	D1_SKCM	26997480	SKCM (Skin Cutaneous Melanoma)	aPD1	15	12
	D2_SKCM	29033130	SKCM (Skin Cutaneous Melanoma)	aPD1	10	39
	D3_SKCM	30753825	SKCM (Skin Cutaneous Melanoma)	aPD1	19	22
	D4_SKCM	32708981	SKCM (Skin Cutaneous Melanoma)	aPD1	8	6
	D5_SKCM	27956380	SKCM (Skin Cutaneous Melanoma)	aCTLA4	8	16
	D6_KIRC	32472114	KIRC (Kidney Renal Clear Cell Carcinoma)	aPD1	39	133
	D7_KIRC	29301960	KIRC (Kidney Renal Clear Cell Carcinoma)	aPD1	8	25
	D8_LUAD	32879421	LUAD (Lung Adenocarcinoma)	aPD1	5	11
	D9_LUAD	32762727	LUAD (Lung Adenocarcinoma)	aPD1_aPDL1	8	19
	D10_STAD	30013197	STAD (Stomach Adenocarcinoma)	aPD1	12	33
	D11_BLCA	29443960	BLCA (Bladder Urothelial Carcinoma)	aPDL1	68	230
	D12_GBM	30742119	GBM (glioblastoma)	aPD1	9	7
validation cohorts	V1_SKCM	29170503	SKCM (Skin Cutaneous Melanoma)	Adoptive T-cell therapy (ACT)	10	15
	V2_SKCM	30127394	SKCM (Skin Cutaneous Melanoma)	aPD1_aCTLA4	2	9
	V3_UC	34294892	UC (Urothelial Cancer)	aPD1	16	71
	V4_SKCM	28494868	SKCM (Skin Cutaneous Melanoma)	aPD1	2	3
	V5_SKCM	31792460	SKCM (Skin Cutaneous Melanoma)	aPD1	17	24

Table S5. Mouse datasets with gene expression profiles before and after drug treatment. Related to Figure 3.

GEO	Pubmed	Drug1	Drug2	CancerType	Number of samples	SampleType
GSE149825	33589424	birinapant	anti-PD-1/CTLA-4	melanoma (primary)	12	murine melanoma mice (established B16F10 melanoma tumor mouse)
GSE152925	32723834	swainsonine	anti-PD-L1	melanoma	28	murine melanoma mice (Wildtype or Man2a1 knockout B16F10 mice)
GSE120500	30540933	olaparib		ovarian cancer	12	murine overian tumor transplanted mice (Brca1-deficient ovarian tumor bearimg mice)
GSE114601	30087114	JQ1	anti-PD-1	Non-Small-Cell Lung Cancer	8	GEMM mice (Krasþ/LSL-G12D; Trp53L/L (KP) NSCLC mouse models)
GSE160785	34031121	celecoxib	anti-PD-1	colorectal cancer	49	murine CT26 colorectal carcinoma mice (CT26 tumor Balb/c mice)

Table S8. Eight candidate compounds that may enhance the efficacy of immunotherapy for liver cancer. Related to Figure 5.

compound	MoA	FDA approved drugs	dataset	rank	LIHC_BS
CGP-60474	CDK inhibitor	no	GSE70138	3	9.021119807
PD-0325901	MEK inhibitor	no	GSE70138	4	8.586601074
trametinib	MEK inhibitor	yes	GSE70138	7	6.655274543
tyrphostin-AG-494	EGFR tyrosine kinase inhibitor	no	GSE92742	1	19.78885899
phenothiazine	Dopamine D2 receptor antagonist	yes	GSE92742	2	17.79438242
docetaxel	Tubulin inhibitor	yes	GSE92742	4	17.32441355
tubacin	HDAC inhibitor	no	GSE92742	5	17.19915011
SB-366791	TRPV1 antagonist	no	GSE92742	7	16.68389834

Note: due to less known or diifficult to obtain, compounds named only with 'BRD-' was removed from GSE92742 for selecting candidate compounds.

Table S9. Clinicopathologic features of patients for PDOTs constructions. Related to Figure 5.

# experiments of 8 compounds listed in Table S8.

hepatocellular carcinoma	Patient 1	Patient 2	Patient 3
Age,years	88	53	78
Sex	Male	Male	Female
HBsAg, COI	(-)0.274	(+)1722	(+)1677
HBV DNA, IU/mL	< 20	< 20	$1.34*10^{7}$
CNLC stage	Ib	Ib	Ia
BCLC stage	A	A	A
Child-Pugh	A	A	A
AFP, ng/mL	99.4	637	13.2
PIVKA-II, mAU/mL	9137	4959	378
Diameter of intrahepatic tumors, cm	6.8	6.5	4

Abbreviation: HBsAg, hepatitis B surface antigen; CNLC, China liver cancer; BCLC, Barcelona Clinic liver cancer; AFP, alpha-fetoprotein.

# experiments of mitoxantrone

hepatocellular carcinoma	Patient 1	Patient 2	Patient 3
Age,years	77	71	69
Sex	Male	Male	Male
Race	East Asian	East Asian	East Asian
HBsAg, COI	(+)9.96	(-)0.482	(+)4.36
HBV DNA, IU/mL	< 20	< 20	< 20
CNLC stage	Ⅰ A	Ⅰ A	Ⅰ IB
BCLC stage	A	A	B
Child-Pugh	A	A	A
AFP, ng/mL	105.3	376	59.6
PIVKA-II, mAU/mL	222	460	33
Diameter of intrahepatic tumors, cm	64.2	4.5	5

# experiments of SB-366791 and mitoxantrone

colon cancer	Patient 1	Patient 2	Patient 3
Age,years	67	80	57
Sex	Male	Male	Female
Race	East Asian	East Asian	East Asian
Tumor sidedness	Right	Left	Right
Tumor stage	IIIA	IIIB	IIIA

# experiments of SB-366791 and mitoxantrone

lung adenocarcinoma	Patient 1	Patient 2	Patient 3
Age,years	61	59	49
Sex	Male	Male	Female

Race	East Asian	East Asian	East Asian
Tumor stage	IIB	IIA	IIA
