# Supplementary material for: Computational framework for prioritizing candidate compounds overcoming the resistance of pancancer immunotherapy
Source: Cell Rep Med. 2025 Aug 5;6(8):102276. doi: 10.1016/j.xcrm.2025.102276 (PMC12432370; doi:10.1016/j.xcrm.2025.102276)
Supplement: Document S2. Article plus supplemental information [file mmc8.pdf]

Cell Reports Medicine

Article

Computational framework for prioritizing candidate compounds overcoming the resistance of pancancer immunotherapy

Graphical abstract

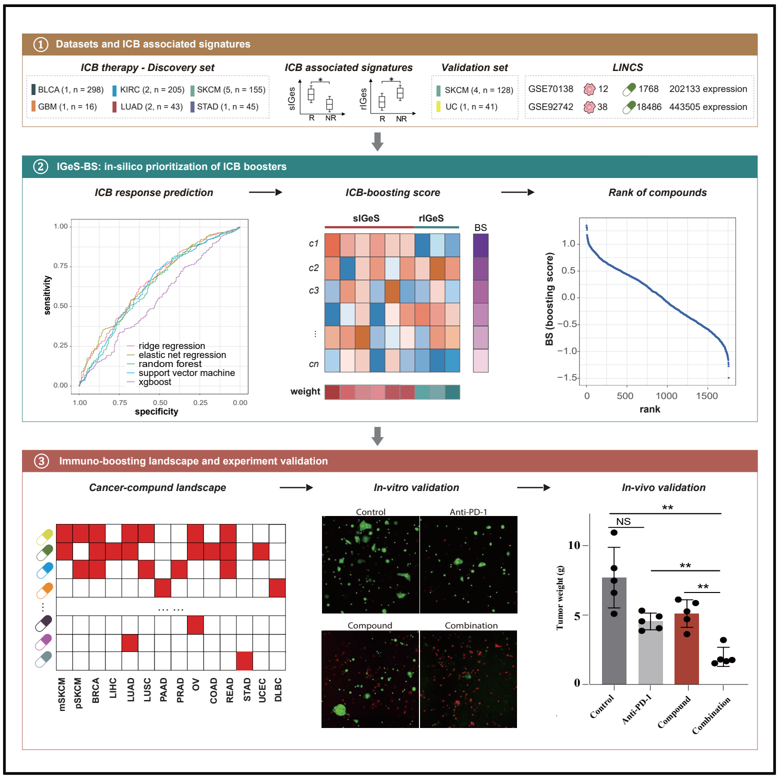

Authors

Fangyoumin Feng, Tian He, Ping Lin, ..., Jia Fan, Bo Hu, Hong Li

Correspondence

hu.bo@zs-hospital.sh.cn (B.H.), lihong01@sinh.ac.cn (H.L.)

In brief

Feng et al. develop a computational framework (IGeS-BS) to recommend compounds that enhance immunotherapy responses and generate an immuno-boosting landscape for more than 10,000 compounds and 13 cancer types. Further experiments prove that two compounds could largely alleviate the resistance to anti-PD1 therapy in liver cancer.

Highlights

- Pancancer analysis identifies consistent and predictive immunotherapy-related signatures
- IGeS-BS is developed for prioritizing immunotherapy booster
- IGeS-BS shows excellent performance in computational and experimental validation
- SB-366791 and CGP-60474 boost the efficacy of immunotherapy in liver cancer

Feng et al., 2025, Cell Reports Medicine 6, 102276
August 19, 2025 © 2025 The Author(s). Published by Elsevier Inc.
https://doi.org/10.1016/j.xcrm.2025.102276

Cell Reports Medicine

CellPress
OPEN ACCESS

Article

Computational framework for prioritizing candidate compounds overcoming the resistance of pancancer immunotherapy

Fangyoumin Feng,[1,4] Tian He,[2,4] Ping Lin,[1,4] Jinwu Hu,[3,4] Bihan Shen,[1] Zhixuan Tang,[1] Jian Zhou,[2] Jia Fan,[2] Bo Hu,[2,*] and Hong Li[1,5,*]
[1]Shanghai Institute of Nutrition and Health, University of Chinese Academy of Sciences, Chinese Academy of Sciences, Shanghai 200031, China
[2]Department of Hepatobiliary Surgery and Liver Transplantation, Liver Cancer Institute, Zhongshan Hospital, Fudan University, and Key Laboratory of Carcinogenesis and Cancer Invasion, Ministry of Education, Shanghai 20032, China
[3]Department of Liver Cancer, Shanghai Geriatric Medical Center, Shanghai 20032, China
[4]These authors contributed equally
[5]Lead contact
*Correspondence: hu.bo@zs-hospital.sh.cn (B.H.), lihong01@sinh.ac.cn (H.L.)
https://doi.org/10.1016/j.xcrm.2025.102276

SUMMARY

Combination therapy has emerged as an effective approach to overcome resistance to immunotherapy. However, only a small number of drugs have been identified with synergistic effects with immunotherapy. Here, we develop a computational framework (IGeS-BS) to recommend compounds that potentially overcome resistance to immunotherapy. A meta-analysis of approximately 1,000 transcriptomes from immunotherapy patients revealed 33 tumor microenvironment (TME) signatures that can robustly and accurately estimate immunotherapy responses. An immuno-boosting landscape for more than 10,000 compounds and 13 cancer types was subsequently generated on The Cancer Genome Atlas (TCGA) and The Library of Integrated Network-Based Cellular Signatures (LINCS) datasets. Furthermore, the immuno-boosting effects of several high-scoring compounds were evaluated by *in vitro* and *in vivo* experiments in hepatocellular carcinoma and other cancer types. The results showed that the two best compounds (SB-366791 and CGP-60474) significantly alleviate the resistance of hepatocellular carcinoma to anti-PD1 therapy by activating immune cells. Collectively, our research provides an efficient framework for discovering compounds that enhance immunotherapy responses.

INTRODUCTION

Immunotherapy, especially immune checkpoint blocking (ICB) therapy, is an effective tumor treatment strategy that is superior to conventional methods.[1–3] Although ICB therapy has led to significant clinical improvement, some patients suffer primary resistance or acquired resistance due to the high heterogeneity of cancer cells and the complexity of the immune microenvironment.[4,5] Therefore, combination treatment involving ICB and other anticancer drugs has received increasing attention and has become an important research direction for overcoming resistance to immunotherapy. Many clinical trials have focused on the combination of ICB therapy with chemotherapy or targeted therapy. For example, the combination of the angiogenesis inhibitor lenvatinib and anti-PD1/PDL1 drugs has shown superior synergistic effects in liver cancer.[6,7] However, drug candidates in most clinical trials rely on expert experience and prior knowledge. If computational analysis can predict synergistic drugs for ICB, it may decrease the time and increase the likelihood of discovering new drug combinations. Therefore, a systematic and comprehensive understanding of the molecular characteristics related to tumor immunity and the development of computational methods to recommend immunotherapy combination strategies are urgently needed.

Recently, comparative studies of immunotherapy responder and nonresponder populations have identified a number of molecular markers and several prediction models for predicting the efficacy of immunotherapy. PDL1 protein expression,[8,9] tumor mutation burden,[10,11] microsatellite instability, and defects in DNA mismatch repair[10,12,13] have been approved by the Food and Drug Administration (FDA) as biomarkers for ICB therapy. In addition, markers of cytotoxic T lymphocytes (*CD8A* and *CD8B*)[14] and interferon-gamma-related (IFNG) gene sets (including interferon gamma *IFNG*, *STAT1*, *IDO1*, *CXCL10*, *CXCL9*, and major histocompatibility complex, class II, DR alpha, *HLA-DRA*) have also been reported to be associated with immunotherapy efficiency.[14,15] The Immunophenoscore model (IPS) predicts responses to immunotherapy through the weighted integration of 160 immune-related gene sets, such as those related to antigen presentation, immune checkpoints and regulators, immune effector cells, and suppressor cells.[8] The tumor immune dysfunction and exclusion (TIDE) algorithm

© 2025 The Author(s). Published by Elsevier Inc.
This is an open access article under the CC BY license (http://creativecommons.org/licenses/by/4.0/).

constructs signatures of T cell dysfunction and exclusion and integrates them to determine whether the tumor responds to immunotherapy.[16] Another study defined a 15-gene tumor immunological phenotype (TIP) signature distinguishing "cold" tumors from "hot" tumors and then predicted immunotherapy responses on the basis of their expression patterns.[17]

Relative to single immunotherapy, the development of computational methods for prioritizing immunotherapy combination strategies is still in a preliminary stage. An intuitive strategy is to determine the number of cancer cells and infiltrating immune cells and the inhibitory concentration of drugs used in drug treatment experiments in mouse models and then construct differential equations to estimate the efficacy of combining ICB with other drugs.[18–20] Another strategy is to construct sensitive and resistant mouse models, analyze differential network modules between two states, find potential drug-targeted genes from the differential modules, and finally evaluate the synergistic anticancer effects of drug combinations via *in vivo* experiments.[21] However, these strategies are not economically feasible for large-scale screening of drugs and compounds for multiple cancer types. Currently, a data-driven method named CM-Drug enables the screening of compounds that may enhance the efficacy of ICB treatment based on gene sets associated with ICB responses.[22] A few studies have investigated the molecular characteristics of the "cold" and "hot" states of the tumor microenvironment (TME), identified features related to "cold" tumors, and then inferred and validated drugs that might relieve immune suppression,[17,23,24] suggesting the potential to predict the combination of immunotherapy and other drugs by regulating the TME.

The bottlenecks in the prediction of combinations of ICB and other drugs include the complex interactions between the immune microenvironment and tumor cells, which involve multiple genes and pathways, the lack of response data for drug combination treatment in patients and animal models, and the lack of mature methods for measuring the impacts of other drugs on immunotherapy. Therefore, we propose a feasible strategy to develop methods to identify immunotherapy-related TME signatures and quantify the effects of chemotherapy or targeted drugs on immunotherapy via their regulation of the TME.

Here, we developed a computational framework for enhancing immunotherapy and discovered compounds that overcome immunotherapy resistance in pancancer. First, we systematically characterized the differences in the TME between responders and nonresponders to immunotherapy and constructed a weighted model of 33 immunotherapy-related gene expression signatures (IGESs) that could predict immunotherapy response. Next, we developed a computational strategy to quantify compound-induced TME changes and prioritized compounds with the potential to increase the efficacy of immunotherapy. Finally, *in vitro* and *in vivo* experiments validated the effects of the candidate compounds on overcoming resistance to pancancer immunotherapy.

RESULTS

Pancancer analysis identified consistent and predictive immunotherapy-related signatures

To obtain consistent and predictive signatures associated with immunotherapy efficacy across cancers (Figure 1A), we initially collected 371 TME-related gene sets (TME_GS) manually and subsequently evaluated the association between TME_GS and immunotherapy efficacy via meta-analysis of 12 cohorts (the discovery sets), selected the optimal signature combination, and ultimately validated them in another 5 independent cohorts (validation sets).

The 371 TME_GS were divided into three categories: 39 gene sets used in previously published immunotherapy response predictors, 128 gene sets reported to be anti-tumor or protumor, and 204 gene sets highly expressed in tumor-infiltrating cells (Table S1). The 12 cohorts with transcriptomic data and the clinical response to immunotherapy included 762 patient samples from 6 types of solid tumors (5 with melanoma, 2 with clear cell renal cell carcinoma, 2 with lung adenocarcinoma, 1 with stomach adenocarcinoma, 1 with glioblastoma, and 1 with urothelial carcinoma; Table S2). By scoring each sample with TME_GS and comparing the differences between responders and nonresponders in every cohort, we found notable cohort variations due to the complex mechanism of immunotherapy and high patient heterogeneity. A meta-analysis of 12 cohorts revealed that 130 TME_GS were significantly related to immunotherapy response; these TME_GS were referred to as IGeSs ($p < 0.05$; Figures 1B; Table S3). Compared with single-cohort studies, meta-analyses could refine IGeSs with contradictory results in multiple cohorts.

Among the 130 IGeSs, 117 signatures with odds ratios (ORs) greater than 1 were associated with immunotherapy sensitivity (sIGeS, Figure 1B, left part), whereas 13 signatures with ORs less than 1 were associated with immunotherapy resistance (rIGeS, Figure 1B, right part). Our results confirmed previously known associations with immunotherapy. For example, interferon gamma pathway (IFNG)-related (OR = 1.69 [1.18, 2.42], $p = 0.004$) and PD1-related terms (OR = 1.62 [1.12, 2.36], $p = 0.011$) are sIGeSs and T cell exclusion (OR = 0.68 [0.53, 0.87], $p = 0.0026$)- and cancer-associated fibroblasts (OR = 0.76 [0.59, 0.99], $p = 0.0488$) are rIGeS. Most immune cells were sIGeSs, such as CD8$^+$ T cells (OR = 1.75 [1.16, 2.65], p value = 0.0079), effector memory CD8$^+$ T cells (OR = 1.45 [1.08, 1.96], p value = 0.014), T helper type 1 (OR = 1.63 [1.21, 2.20], $p = 0.0015$), and natural killer cells (OR = 1.55 [1.10, 2.18], $p = 0.012$). Macrophages are more complex, and their different states may be inversely associated with immunotherapy. M1 macrophages are significantly related to immunotherapy sensitivity (OR = 1.41 [1.05, 1.89], $p = 0.0219$), which is consistent with their proinflammatory role, whereas M2 macrophages are strongly associated with immunotherapy resistance (OR = 0.66 [0.51, 0.86], $p = 0.0022$).

Because of the small number of genes per IGeS and the obscure matched cancer type, we conducted an expansion strategy to make IGeS detectable and robust in tissues and cell lines with pancancer entities (STAR Methods). These extended signatures reflect the scores of the original signatures well (Figure S1). Owing to the potential correlation among IGeSs, we explored the optimal combination of IGeSs that could estimate the response to immunotherapy. Five methods with less impact of multicollinearity were used to construct classification models to distinguish responders and nonresponders from 5 validation cohorts, including elastic net regression (EN), ridge regression (RR), random forest (RF), support vector machine

CellPress
OPEN ACCESS

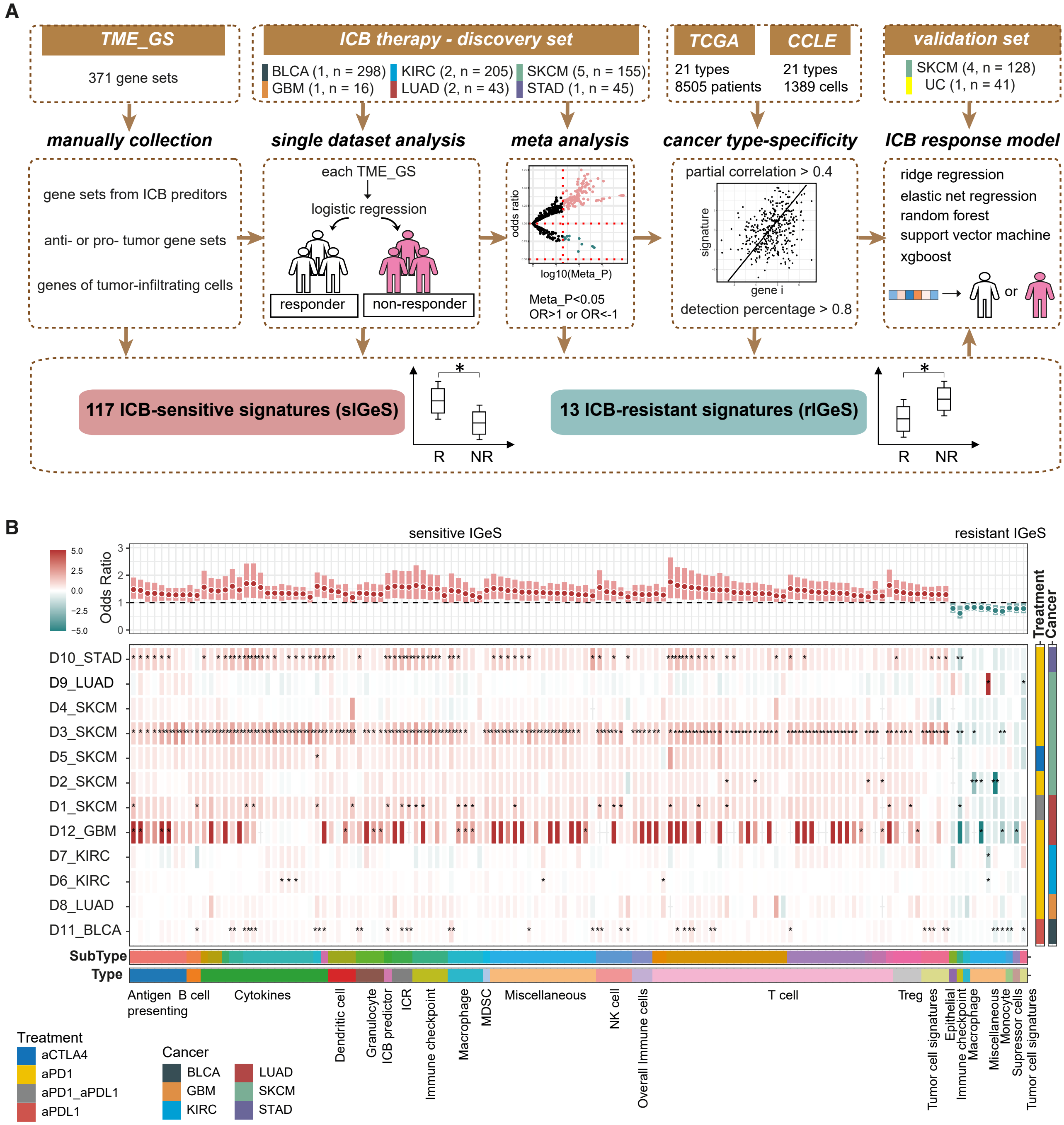

Figure 1. Meta-analysis identified consistent signatures of pancancer immunotherapy

(A) Workflow for integrating pancancer immunotherapy datasets and identifying signatures associated with immunotherapy efficacy.

(B) Heatmap illustrating the immunotherapy-related gene expression signatures (IGeSs). IGeS was selected through the integrated analysis of differential gene sets between responders and nonresponders across 12 immunotherapy cohorts (Meta_P < 0.05). Each row corresponds to a cohort dataset, and each column represents an IGeS. The color indicates the odds ratio (OR) of IGeS scores between responders and nonresponders. IGeS with OR > 1 or OR < 1 are named sensitive IGeS (red) and resistant IGeS (green), respectively. Error bars represent the confidence intervals derived from the random effects model in the meta-analysis. Asterisks (*$p < 0.05$ and **$p < 0.01$) indicate statistical significance for coefficient p values derived from each logistic regression model.

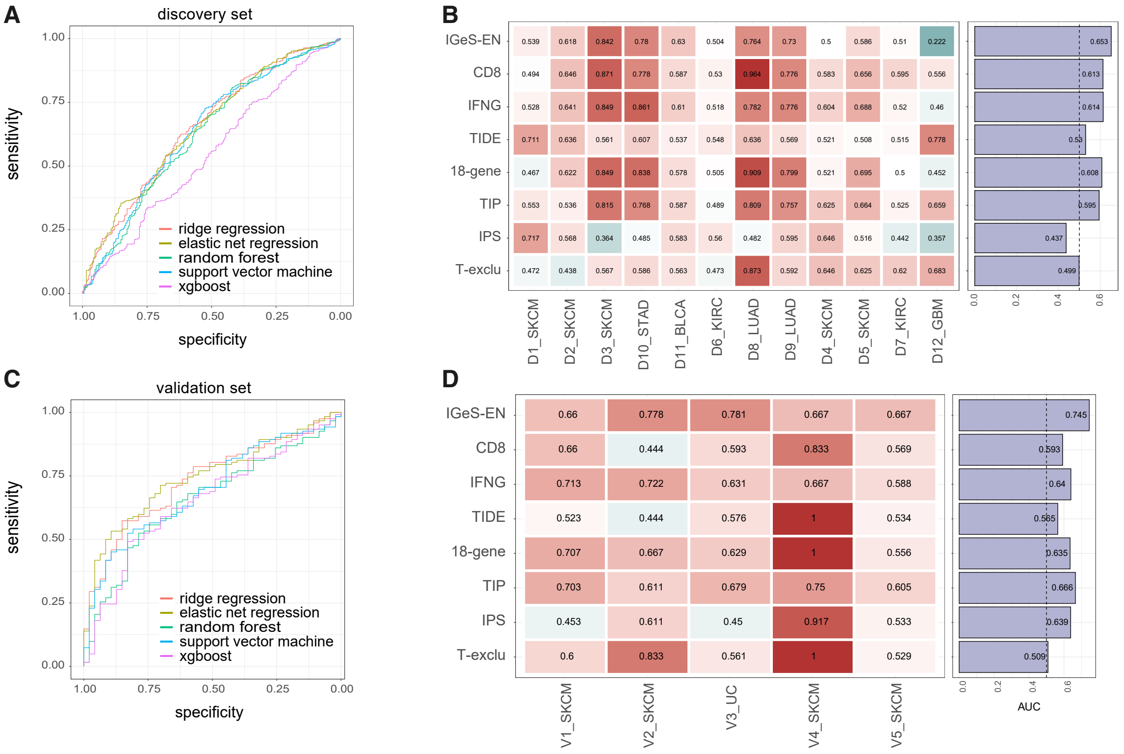

Figure 2. The IGeS could predict the response to pancancer immunotherapy

(A) Receiver operating characteristic (ROC) curves showing the prediction performances of different models in the discovery sets.

(B) Comparison between IGeS-EN and other published signatures for predicting the efficacy of immunotherapy. The values in the heatmap are the area under the curve (AUC) values obtained from the discovery sets.

(C and D) Similar to (A) and (B) for another 5 cohorts in the validation sets. The barplot on the right panel shows the overall AUC computed across all cohorts.

(SVM), and the gradient boosted tree algorithm (XGBoost). 5-fold cross-validation of the integrated discovery sets revealed that elastic net regression (IGeS-EN) achieved the best performance, with an overall area under the curve (AUC) of 0.653 (Figures 2A and 2B). For the validation set, IGeS-EN had the best performance, with an overall AUC of 0.745 (Figures 2C and 2D). The regularization of elastic net regression can perform automatic variable selection. Thirty-three IGeSs (26 sIGeSs and 7 rIGeSs) were selected by IGeS-EN, which reflected the importance of each IGeS while limiting the redundancy (Table S4). Compared with seven published prediction methods in validation sets, IGeS-EN achieved comparable performance in all cohorts (Figure 2D), indicating that the combination of 33 IGeS achieved robust and accurate estimation of pancancer immunotherapy efficacy.

In silico screening of immunotherapy boosters

We hypothesized that a small-molecule compound might have the potential to increase immunotherapy efficacy if it can significantly upregulate sIGeS but significantly downregulate rIGeS. On the basis of this hypothesis, we developed a computational framework (IGeS-BS) to prioritize compounds for boosting immunotherapy (Figure 3A). First, gene expression profiles before and after compound perturbation were obtained from high-throughput perturbation experiments on cancer models. Second, a perturb gene expression score (PGES) was defined to quantify the significant effects of a compound on the expression change of each IGeS in cancer level. Third, the PGES values were weighted based on IGeS-EN feature weights and summed up to obtain an ICB-boosting score (BS) for each compound, which reflected the potential of a compound to increase immunotherapy efficacy. Fourth, all drugs/compounds were ranked on the basis of the BS for each cancer type.

Owing to the relatively high cost and low efficiency of animal models, large-scale perturbation experiments are generally produced from cancer cell lines. Therefore, we utilized one of the largest cell line perturbation databases, LINCS, to implement the proposed computational framework. To evaluate whether the PGES calculated from the LINCS cell lines could recapitulate the *in vivo* expression changes, we collected five mouse datasets with gene expression profiles before and after drug treatment (Table S5). The consistency ratio of drug-induced IGeS expression changes between mice and cell lines ranged from 84.62% to 100% (Figure S2). Therefore, it is reasonable to apply LINCS to IGeS-BS for large-scale screening of compounds for multiple cancer types.

After applying two LINCS datasets (GEO: GSE70138 and GSE92742) to the IGeS-BS framework, a cancer-compound landscape was generated to present the potential of boosting immunotherapy (Table S6). It is more possible for compounds

CellPress
OPEN ACCESS

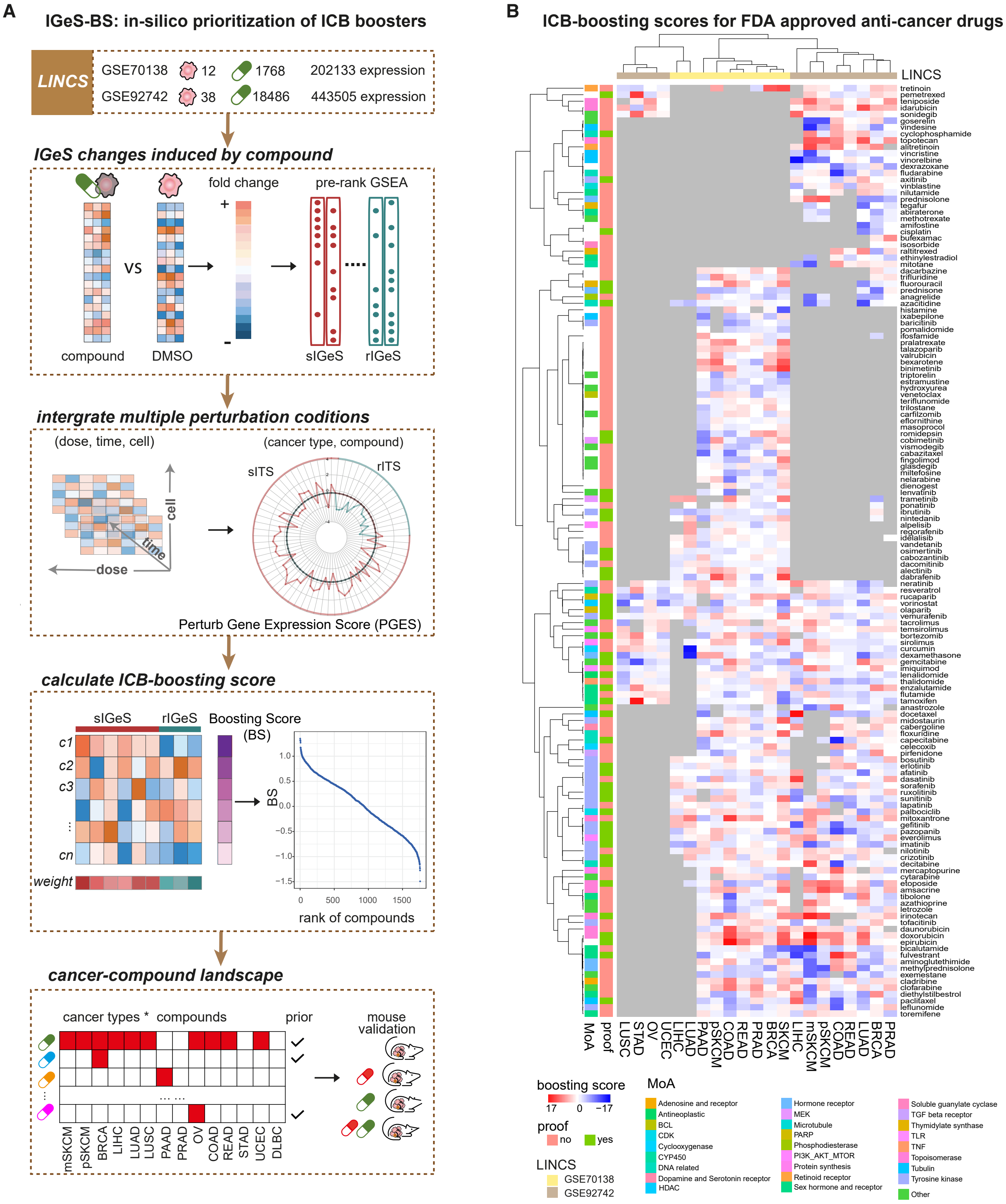

(legend on next page)

with higher positive BSs to boost immunotherapy and combine with ICB to produce synergistic effects. Some compounds had higher scores in multiple cancer types, implying that they might improve immunotherapy efficacy in a pancancer manner, whereas others showed obvious differences among cancer types, suggesting their roles in a type-specific way. The BS of 143 FDA-approved drugs for 13 cancer types (Figure 3B) revealed several known immunotherapy boosters, such as dabrafenib (rank = 8), trametinib (rank = 66), and vemurafenib (rank = 80) for metastatic skin cutaneous melanoma (mSKCM); dabrafenib (rank = 4), cobimetinib (rank = 49), and vemurafenib (rank = 130) for primary skin cutaneous melanoma (pSKCM); cediranib (rank = 117) and olaparib (rank = 328) for colon adenocarcinoma (COAD); and vorinostat (rank = 106) for prostate adenocarcinoma (PRAD) in GEO: GSE70138. Interestingly, some chemotherapies, such as fluorouracil and irinotecan, have also shown potential for enhancing immunotherapy for various cancer types such as COAD and rectum adenocarcinoma (READ).

IGeS-BS could identify known immunotherapy boosters

To validate the IGeS-BS strategy, we first manually curated known immunotherapy combinations in different cancer types (Table S7), including 19 FDA-approved combinations and 258 combinations in clinical trials (87 of which were in phase 3 trials). For each cancer type, the IGeS-BS scores of known immunotherapy boosters were compared with the scores of other compounds. Known boosters had significantly greater BSs in seven cancer types: pSKCM, mSKCM, liver hepatocellular carcinoma (LIHC), PARD, READ, breast invasive carcinoma (BRCA), and COAD (Figures 4A–4C, effect size >1 and $p < 0.05$). This indicates that the IGeS-BS strategy can find known boosters.

We further utilized data from drug combination experiments conducted on mouse models to validate the predictive performance of the IGeS-BS and to explore the underlying mechanisms of drug boosting as reflected by IGeSs. The first example is birinapant, which has been reported to enhance the ICB response in primary melanoma by increasing the activity of major histocompatibility complex (MHC) class I.[25] Birinapant ranks 20th (top 1.2% in GEO: GSE70138) on the basis of the BSs calculated by the IGeS-BS in pSKCM (Figure 4D). IGeS-BS profiling of pSKCM revealed that 80% of the sIGeS (21/26) were upregulated under birinapant therapy, including the MHC signature (Figure 4E). The expression profiles of melanoma mice treated with vehicle or birinapant and/or ICB were downloaded from GEO: GSE149825. The MHC signature score indeed increased after birinapant treatment in the mice (Figure 4F), which is highly consistent with the reported synergistic mechanism of birinapant and ICB.[25] The second example is olaparib, for which both the prediction of IGeS-BS (Figure 4G) and mouse experiments have shown better effects of olaparib combined with ICB.[26] The results from the IGeS-BS prediction revealed that olaparib mainly upregulated sIGeS, including IFN gamma (sIGeS7), PD1 and T cell receptor signaling (sIGeS11), and dendritic cells (sIGeS24) (Figure 4H). The measured mouse expression profiles also demonstrated that the scores of sIGeS11 and sIGeS24 increased after olaparib treatment, which is consistent with the IGeS-BS prediction (Figure 4I).

Predicted immunotherapy booster for hepatocellular carcinoma

Furthermore, liver cancer was used as a scenario to validate the effects of predicted immunotherapy boosters via *in vitro* and *in vivo* treatment experiments. The top 20 compounds estimated by IGeS-BS from the two LINCS expression sets are shown in Figure 5A. Candidate compounds were selected on the basis of the following strategies: their ICB-BS is positive and ranks in the top 20 in GEO: GSE70138 or GSE92742 datasets, their mechanism of action (MoA) has already been determined, and some known immune boosters have the same MoA. This yields eight compounds (Table S8), including three FDA-approved drugs (phenothiazine, docetaxel, and trametinib) that are more likely to be applied in clinical practice and five other compounds. We first determined the half maximal inhibitory concentration (IC_{50}) of these eight compounds in the Huh7 and Hep3B cell lines (Figures 5B and S3A). According to these IC_{50} values, we further conducted experiments using patient-derived organotypic tumor spheroids (PDOTs) in a microfluidic culture device as previously reported.[27,28] Three surgically removed samples obtained from hepatocellular carcinoma (HCC) patients resistant to anti-PD1 therapy were utilized to conduct PDOTs (Table S9). Multicolor immunofluorescence staining shows that samples from these three resistant patients had significantly lower PDL1 and PD1 expression and few $CD3^+$ and $CD8^+$ T cells compared to those from partial-response patients (Figure S4). Then PDOTs were cultured under 18 conditions: the control group, the group treated with the anti-PD1 antibody alone, groups treated with each of the eight predicted compounds, and groups subjected to the combination treatments involving the anti-PD1 antibody and another compound. The combination therapy demonstrated significantly greater efficacy in killing tumor cells (Figures 5C and S3B), suggesting that all eight compounds exhibited a certain capacity to overcome resistance to immunotherapy. Among all the selected compounds, SB-366791 and CGP-60474 had the most significant effects (Figure 5D).

SB-366791 and CGP-60474 overcome resistance to immunotherapy

Based on the results of PDOTs examination, we further validated the function of SB-366791 and CGP-60474 in a spontaneous tumorigenesis HCC mouse model. Drug therapy was taken

Figure 3. A computational framework for prioritization of immunotherapy-boosting compounds

(A) Flowchart depicting the process of ranking compounds.

(B) Pancancer landscape of ICB-boosting scores for FDA-approved anticancer drugs. Each row represents a drug, and each column represents a cancer type available in the LINCS datasets (GEO: GSE70138 and GSE92742). The color indicates the ICB-boosting scores calculated by the IGeS-BS framework. A more intense red color (higher score) means that the compound is more likely to reverse the expression of IGeS to a greater extent, and it is inferred to enhance the efficacy of ICB therapy. Gray indicates that there is no drug-perturbed expression profile for that drug in a particular type of cancer. More results for the other compounds are provided in Table S6.

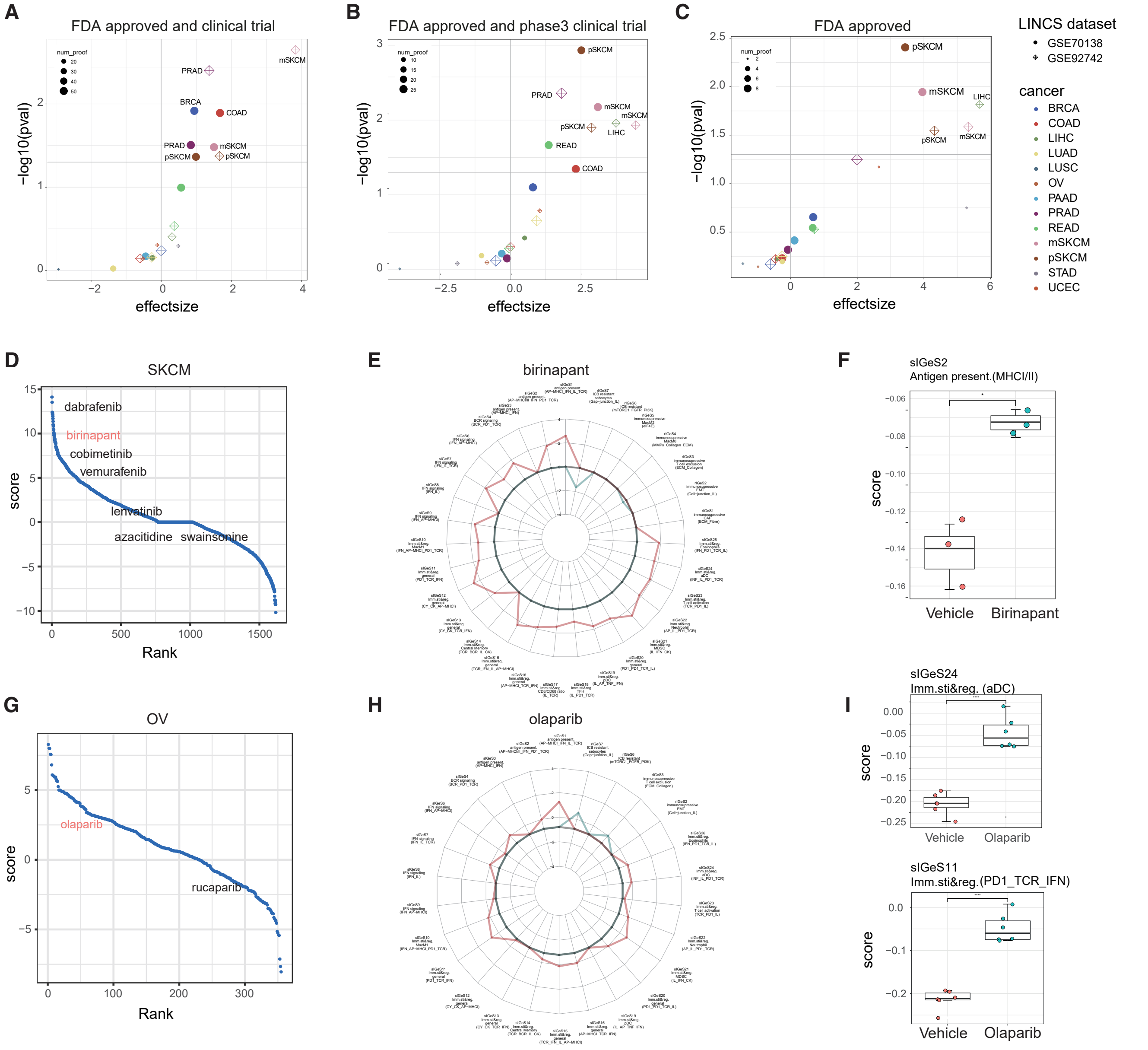

Figure 4. Validation of the accuracy of IGeS-BS prediction

(A–C) Comparisons of the ICB-boosting scores between known immunotherapy boosters and other compounds. (A) Known boosters approved by the FDA or in clinical trials. (B) Known boosters approved by the FDA or in phase 3 clinical trials. (C) Known boosters approved by the FDA. Effect size was calculated from the difference of average boosting score; *p* value was calculated using the Wilcoxon rank-sum test; horizontal line indicates $p = 0.05$.

(D) Rank of compounds based on the ICB-boosting scores of primary skin cutaneous melanoma (SKCM).

(E and H) Radar plots showing each IGeS score for birinapant and olaparib in the LINCS cell line data. Red indicates sIGeS, while green indicates rIGeS.

(F) Changes in MHC signatures after birinapant treatment in melanoma mice. Gene expression profiles were obtained from GEO: GSE149825. *p* values were calculated via t tests. * indicates $p < 0.05$.

(G and I) Similar to (D) and (F), olaparib treatment of ovarian cancer (OV). Gene expression profiles were obtained from GEO: GSE120500. *p* values were calculated via t tests. **** indicates $p < 0.0001$.

14 days after tail-vein injection and was administered every 2 days for a total of 7 times, as shown in Figure 6A. This HCC mouse model was resistant to anti-PD1 treatment, while the combination of anti-PD1 treatment with SB-366791 or with CGP-60474 largely alleviated drug resistance (Figures 6B and 6C, $p < 0.01$). To explore the potential mechanisms involved, the expression profiles of mice subjected to single or combination treatments were compared with those of control mice. There are 355 (296), 145 (47), 838 (693), 195 (129), and 1232 (816) upregulated genes after treatment with the anti-PD1 antibody,

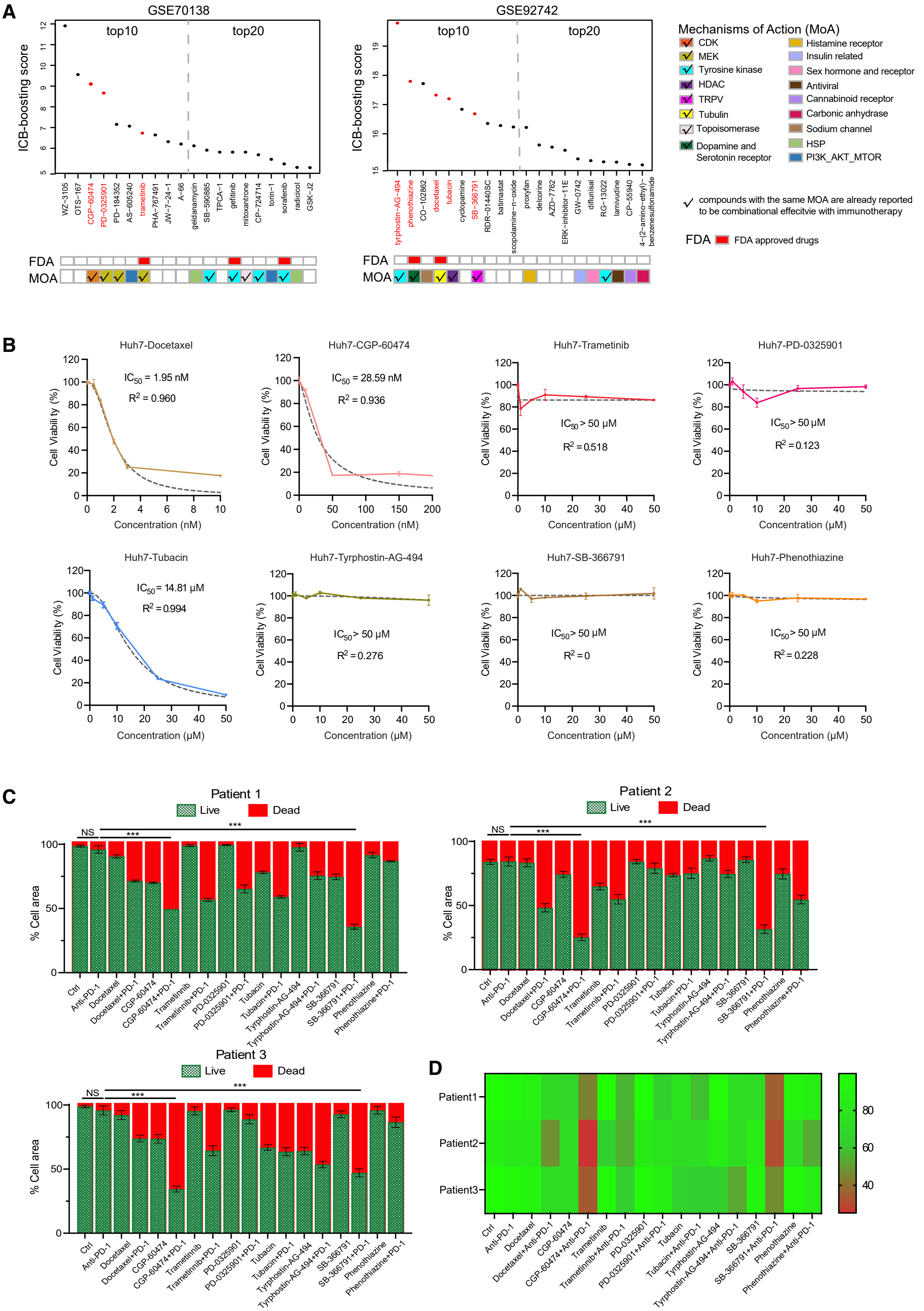

(legend on next page)

CGP-60474, CGP-60474+anti-PD1, SB-366791, and SB-366791+anti-PD1, respectively. Both combinations induced more differentially expressed genes than the single treatments did and resulted in similar functional enrichment. Typically, upregulated genes in the combinations were enriched in T cell activation, whereas downregulated genes were enriched in the cell cycle (Figure 6D). Many genes involved in sIGeS, such as CD8 T cell activation (*Gzmk*), MHC class II (*H2-Aa*, *H2-Ab1*, *H2-DMa*, and *H2-Eb1*), and M1 macrophages (*Ccl5*, *Cxcl9*, and *Cxcl10*), are significantly increased after two types of combination therapies (Figures 6F–6G). Similarly, some genes involved in rIGeS, such as exhausted CD8 T cells (*Dgkh*), are significantly decreased after combination therapy. There are also differences between CGP-60474 and SB-366791 when they are combined with the anti-PD1 antibody. CGP-60474+anti-PD1 increases the expression of some angiogenesis genes (*Vegfa*, *Fgf1*, and *Flt1*); SB-366791+anti-PD1 has stronger effects on immune cell activation by increasing the expression of more immune genes. Additionally, the ratio of M2 macrophages to M1 macrophages was significantly lower in the SB-366791+anti-PD1-treated group, indicating that macrophages are polarized toward the proinflammatory M1 phenotype (Figure 6E). Taken together, the results of both *in vitro* and *in vivo* experiments prove that IGeS-BS identified compounds that can activate immune cells and overcome resistance to liver cancer immunotherapy.

Validation of the IGeS-BS predictions for additional cancer types

With the experimental validation in liver cancer scenario, we further validated the predicted immunotherapy booster of IGeS-BS for additional cancer types. SB-366791 and mitoxantrone were selected since they are predicted to have immune-sensitizing effects on hepatocellular carcinoma, colon cancer, and lung adenocarcinoma (Table S6). Surgical resection samples from three patients with each cancer type served as PDOTs (Table S9). The effects of SB-366791 and its combination treatment have already been validated in hepatocellular carcinoma (Figures 5C and 5D). SB-366791 was further tested in colon cancer and lung adenocarcinoma. The combination of SB-366791 and anti-PD1 treatment had more favorable effects than the single treatments had (Figures S5A–S5C). Mitoxantrone was applied to all three types of tumors, and the combination of mitoxantrone and anti-PD1 had stronger inhibitory effects (Figures S5D–S5F). The efficacy of these two compounds was further verified in subcutaneous mouse models (Figure S6). Although single-drug treatment slightly inhibited tumor growth in some mice, the addition of SB-366791 or mitoxantrone significantly enhanced the antitumor effects of anti-PD1 treatment across all three cancer types.

Benchmark IGeS-BS with other methods

To benchmark IGeS-BS against other similar framework, we further explored whether ICB predictors (such as TIDE, IPS, and TIP) or the recent published CM-Drug method could yield similar findings to IGeS-BS in liver cancer (Table S10). The ICB predictor outputs a score indicating the response to ICB treatment. To assess their potential for predicting ICB-boosting compounds, we calculated the score difference between the compound-treated cancer cells and the DMSO-treated control and summarized results to the cancer level using the same strategy as described in STAR Methods. The benchmark results show that these frameworks can only evaluate compound to some extent. Taking eight experimental validated compounds as examples, TIDE-based framework detected three compounds (CGP-60474, SB-366791, and phenothiazine) with a positive score difference, but only CGP-60474 was ranked in the top 5%; IPS-based model detected six compounds with a positive score difference, but only PD-0325901 was ranked in the top 5%; the TIP-based model identified seven compounds with positive effects, but only three compounds (CGP-60474, SB-366791, and tubacin) were ranked in the top 5%; CM-Drug predicted three of them to have boosting effects, while five of them were filtered out based on CM-Drug's screening strategy ("no effects"). These results further support the advantage of IGeS-BS by introducing more comprehensive immunotherapy-related signatures and a more sophisticated scoring strategy.

DISCUSSION

The present study provides a systematic and comprehensive understanding of immunotherapy-related signatures by integrating the expression profiles of 931 patients from 17 immunotherapy datasets, 8,505 patients from the TCGA pancancer dataset, 19,235 compound-disrupted cell lines from LINCS datasets, and 109 mice from 5 drug-treated datasets. More importantly, a computational framework (IGeS-BS) was constructed to recommend compounds for enhancing the effects of immunotherapy. The performance of IGeS-BS was evaluated via previous known immunotherapy combinations and mouse treatment experiments. Additionally, experiments using PDOTs and mouse models verified the immuno-boosting effects of three compounds (CGP-60474, SB-366791, and mitoxantrone) in three types of cancer.

Considering the lack of microenvironment in cancer cell lines, we extended the IGeS signatures to include genes not only from the TME but also from tumor cells. The resulting IGeS genes are slightly different for each cancer type, making the expression changes estimated from cell lines more suitable for *in vivo* models. Furthermore, IGeS-BS could output cancer

Figure 5. Application of IGeS-BS to liver cancer

(A) Selection of top-ranked compounds for experimental validation.

(B) The IC_{50} results of the selected compounds in the Huh7 cell line. Data are represented as mean ± SD.

(C) Drug test results of PDOTs derived from three anti-PD1-resistant HCC patients. Data are represented as mean ± SD. *p* value was calculated using t test and chi-squared test. *** indicates $p < 0.001$, which shows the comparison of the cell activity between the CGP-60474+PD1 group and the PD1, SB-366791+PD1, and PD1 groups.

(D) Heatmap of three PDOTs' drug test results; the legend shows the relative viability of the PDOTs. Green and red indicate the percentages of live and dead cells, respectively.

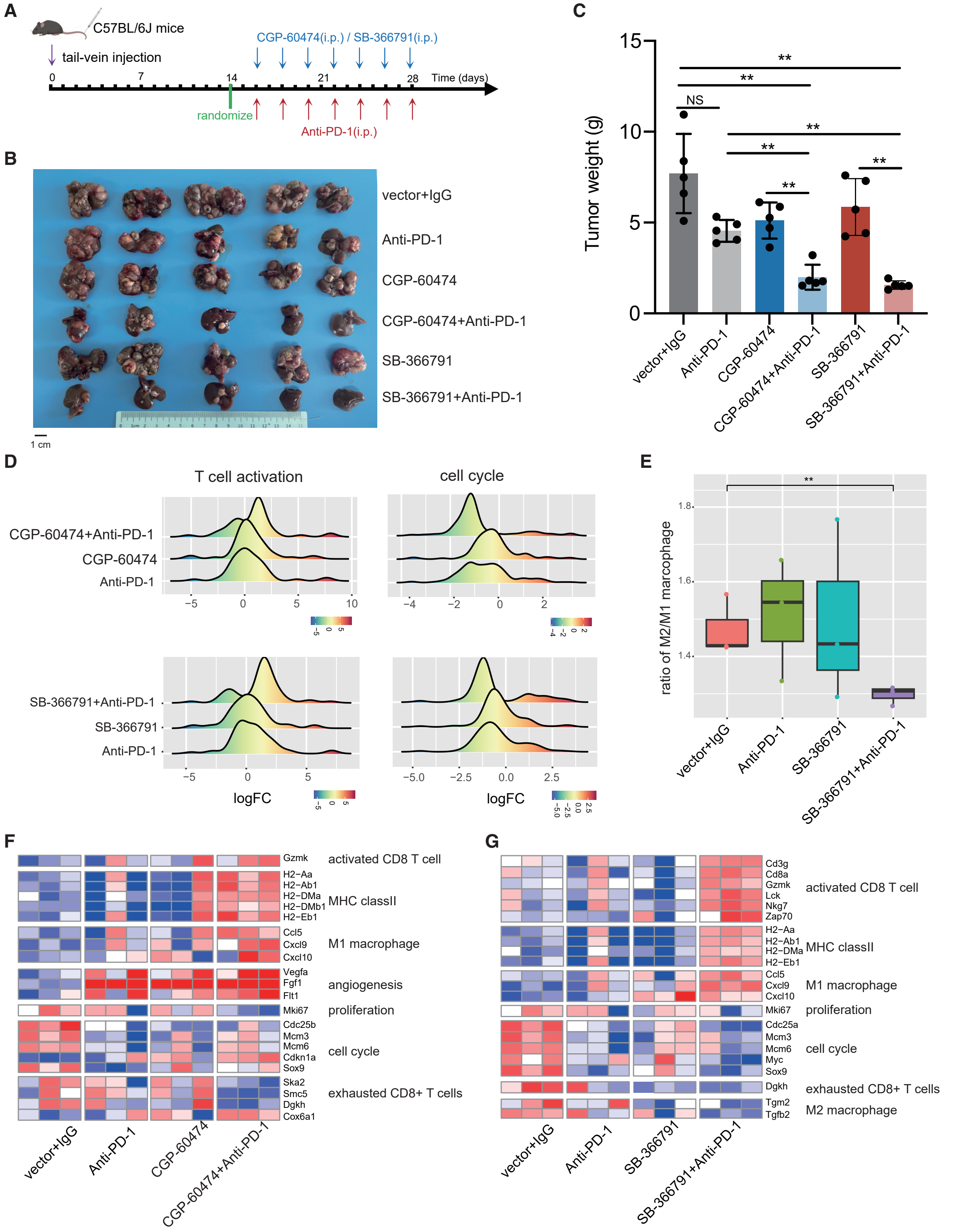

(legend on next page)

type-specific ranks of compounds, helping to understand the common or specific boosters of immunotherapy between cancers. Additionally, the perturbed expression profiles under multiple conditions (varied by dose, time, and cell line) were integrated for each compound. The integrated score is more robust than that from a single condition. The IGeS-BS framework was based on the hypothesis that a compound could boost immunotherapy if it can significantly upregulate immunotherapy-sensitive signatures while downregulate resistant signatures. We attempt to embed other effective ICB predictor such as TIDE, IPS, and TIP in the framework to evaluate compound effects and ultimately prioritize compounds with potential boosting effects; however, the prediction results of other methods in liver cancer scenario are limited.

The basic idea of IGeS-BS seems similar to that of CM-Drug,[22] but IGeS-BS differs significantly from CM-Drug in terms of research objectives, data scale, method details, and application scenarios. The discovery datasets of IGeS-BS cover seven cancer types, and the number of patients is 2.5 times larger than that of CM-Drug. The signatures of the IGeS-BS are more diverse, not only including sensitive signatures but also covering resistance signatures. CM-Drug does not consider the heterogeneity among different cancer types, but the use of cancer-specific IGeS makes IGeS-BS applicable to more cancer types, even for cancers lacking immunotherapy data. The experimental results of liver cancer further proved the power of IGeS-BS for cancers unavailable in the discovery sets.

As data shown in Figure 5, treatment with CGP-60474 alone already inhibited cell viability in PDOTs. To prove that the enhanced anti-tumor effect of CGP-60474 is truly attributed to its sensitization of immunotherapy rather than just a direct effect, we established subcutaneous tumors in BALB/c nude and C57BL/6J mice to test the effects of CGP-60474 alone. Treatments with CGP-60474 (10 mg/kg) were administered intraperitoneally every 2 days and seven times in total. The results showed that CGP-60474 did have certain anti-tumor function ($p < 0.05$) in BALB/c nude mice; however, such function was significantly more obvious in C57BL/6J mice ($p < 0.001$) (Figure S7). Therefore, CGP-60474 could interact with immune system and sensitize immunotherapy.

To further understand the potential mechanisms underlying the synergistic effects of CGP-60474 and SB-366791 in combination with anti-PD1 therapy, we combined the treatment-induced expression profiles and previous literature to infer the potential mechanisms underlying their synergistic effects with anti-PD1 therapy. CGP-60474 is an inhibitor of cyclin-dependent kinases (CDK), which arrests the cell cycle at the G1/S phase and thereby inhibits cell proliferation.[29] The combination of the PD1 inhibitor and CGP-60474 led to the downregulation of genes related to the cell cycle (Figure 6D). Additionally, cell-cycle arrest of tumor cells may alter the profiles of cytokines and chemokines in the TME,[30] attracting more immune cells, such as T cells, to infiltrate the tumor site (Figure 6F), which further exerts synergistic killing effects. SB-366791 is a p38 mitogen-activated protein kinase inhibitor. Inhibiting the p38 signaling pathway may reprogram macrophages from the M2 phenotype to the M1 phenotype.[31] On the other hand, PD1 inhibitors can upregulate the expression of M1 macrophage-related markers, promoting the activation and functional exertion of M1 macrophages.[32] Therefore, the combination of the PD1 inhibitor and SB-366791 may lead to a remarkable increase in the antigen-presenting and tumor-killing abilities of M1 macrophages, thereby producing synergistic effects.

Taken together, IGeS-BS is a powerful method for identifying compounds or drugs that can enhance the efficacy or overcome the resistance of immune checkpoint inhibitors.

Limitations of the study

Despite promising results, our study has limitations. First, IGeS-BS was just compared with other methods in the scenario of liver cancer. More rigorous benchmarking is necessary when more gold-standard datasets are available. Second, the IGeS-BS framework may achieve more accurate prediction with the increase of more compound-perturbed transcriptomes. Additionally, IGeS-BS calculated TME scores from bulk transcriptome data, overlooking the heterogeneity of cancer. With the accumulation of single cell RNA sequencing (scRNA-seq) data, we hope to improve IGeS-BS in the future by integrating scRNA-seq and bulk RNA sequencing. This integration may help researchers better dissect cell subpopulations that are key for immunotherapy resistance, identify subpopulations affected by compounds, and finally infer targets that may overcome immunotherapy resistance by reprogramming the TME.

RESOURCE AVAILABILITY

Lead contact

Further information and requests should be directed to and fulfilled by the lead contact, Hong Li (lihong01@sinh.ac.cn).

Materials availability

This study did not generate any new unique reagents.

Data and code availability

- The data reported in this study have been deposited in the NODE (OEP00006029) and Zenodo (https://doi.org/10.5281/zenodo.15259448) databases.
- The relevant codes used for data analysis are available in a repository at Zenodo (https://doi.org/10.5281/zenodo.15787405) and in a mirroring repository at GitHub (https://github.com/LiHongCSBLab/IGeS_BS).

Figure 6. Treatment of mice with CGP-60474 or SB-366791 overcomes resistance to liver cancer immunotherapy
(A) Schematic view of the *in vivo* treatment of mouse models of liver cancer.
(B and C) Comparison of the tumor weights before and after treatment ($n = 5$ per group). p values were calculated via t tests or Welch's t tests when both groups did not have equal standard deviations. ** indicates $p < 0.01$. Scale bar: 1 cm.
(D) Distribution of differentially expressed genes (log2-fold change) in "T cell activation" and "cell cycle" pathways after treatment.
(E) The ratio of average gene expression values of M2 versus M1 macrophages ($n = 3$ per group). ** indicates $p < 0.01$.
(F and G) Examples of genes whose expressions were upregulated or downregulated after combination therapy ($n = 3$ per group). Differential genes between the treatment and control groups were identified by DESeq2 and filtered by "adjusted $p < 0.1$" and "log2-fold change > 1."

- Any additional information required to reanalyze the data reported in this work paper is available from the lead contact upon request.

ACKNOWLEDGMENTS

This research was supported by the National Natural Science Foundation of China (32470707, 32170680, 32300555, and 82473284), the Natural Science Foundation of Shanghai (21ZR1476000), the CAS Youth Innovation Promotion Association (Y2022076), and the Shanghai Sailing Program (22YF1458000).

AUTHOR CONTRIBUTIONS

F.F., B.H., and H.L. contributed to the initial conceptualization and design of the project; F.F. and B.S. collected and processed publicly available data; F. F. developed the computational framework supervised by H.L.; J.Z. and J.F. provided clinical samples; B.H. designed the experiment validation; T.H. and J.H. conducted PDOTs and mouse models and performed treatment experiments; P.L. carried out bioinformatic analysis for in-house-generated mouse expression profiles; F.F., P.L., and Z.T. analyzed the results; F.F., T.H., B.H., and H.L. wrote the manuscript; all authors reviewed the manuscript and provided critical advice.

DECLARATION OF INTERESTS

The authors declare no competing interests.

STAR★METHODS

Detailed methods are provided in the online version of this paper and include the following:

- KEY RESOURCES TABLE
- EXPERIMENTAL MODEL AND STUDY PARTICIPANT DETAILS
  - Animal studies
  - Patient material
- METHOD DETAILS
  - Immunotherapy pancancer cohorts
  - TCGA pancancer cohorts
  - Identification of immunotherapy-related gene expression signatures
  - Construction and evaluation of the immunotherapy response prediction model
  - Compound-induced gene expression datasets
  - Quantification of the effects of compounds on IGeS expression
  - In silico ranking of compounds for increasing immunotherapeutic response
  - Evaluation of the IGeS-BS with prior data and knowledge
  - Experimental validation of IGeS-BS-predicted compounds for liver cancer
- QUANTIFICATION AND STATISTICAL ANALYSIS

SUPPLEMENTAL INFORMATION

Supplemental information can be found online at https://doi.org/10.1016/j.xcrm.2025.102276.

Received: December 15, 2024
Revised: April 28, 2025
Accepted: July 10, 2025
Published: August 5, 2025

REFERENCES

1. Kruger, S., Ilmer, M., Kobold, S., Cadilha, B.L., Endres, S., Ormanns, S., Schuebbe, G., Renz, B.W., D'Haese, J.G., Schloesser, H., et al. (2019). Advances in cancer immunotherapy 2019–latest trends. J. Exp. Clin. Cancer Res. 38, 268.
2. PD-1/PD-L1 Landscape. https://www.cancerresearch.org/scientists/immuno-oncology-landscape/pd-1-pd-l1-landscape.
3. Vaddepally, R.K., Kharel, P., Pandey, R., Garje, R., and Chandra, A.B. (2020). Review of Indications of FDA-Approved Immune Checkpoint Inhibitors per NCCN Guidelines with the Level of Evidence. Cancers (Basel) *12*, 738. https://doi.org/10.3390/cancers12030738.
4. O'Donnell, J.S., Long, G.V., Scolyer, R.A., Teng, M.W.L., and Smyth, M.J. (2017). Resistance to PD1/PDL1 checkpoint inhibition. Cancer Treat Rev. *52*, 71–81. https://doi.org/10.1016/j.ctrv.2016.11.007.
5. Galon, J., and Bruni, D. (2019). Approaches to treat immune hot, altered and cold tumours with combination immunotherapies. Nat. Rev. Drug Discov. *18*, 197–218. https://doi.org/10.1038/s41573-018-0007-y.
6. Personeni, N., Pressiani, T., and Rimassa, L. (2019). Lenvatinib for the treatment of unresectable hepatocellular carcinoma: evidence to date. J. Hepatocell. Carcinoma *6*, 31–39. https://doi.org/10.2147/JHC.S168953.
7. Kato, Y., Tabata, K., Kimura, T., Yachie-Kinoshita, A., Ozawa, Y., Yamada, K., Ito, J., Tachino, S., Hori, Y., Matsuki, M., et al. (2019). Lenvatinib plus anti-PD-1 antibody combination treatment activates CD8+ T cells through reduction of tumor-associated macrophage and activation of the interferon pathway. PLoS One *14*, e0212513. https://doi.org/10.1371/journal.pone.0212513.
8. Charoentong, P., Finotello, F., Angelova, M., Mayer, C., Efremova, M., Rieder, D., Hackl, H., and Trajanoski, Z. (2017). Pan-cancer Immunogenomic Analyses Reveal Genotype-Immunophenotype Relationships and Predictors of Response to Checkpoint Blockade. Cell Rep. *18*, 248–262. https://doi.org/10.1016/j.celrep.2016.12.019.
9. Davis, A.A., and Patel, V.G. (2019). The role of PD-L1 expression as a predictive biomarker: an analysis of all US Food and Drug Administration (FDA) approvals of immune checkpoint inhibitors. J. Immunother. Cancer 7, 278.
10. Boyiadzis, M.M., Kirkwood, J.M., Marshall, J.L., Pritchard, C.C., Azad, N. S., and Gulley, J.L. (2018). Significance and implications of FDA approval of pembrolizumab for biomarker-defined disease. J. Immunother. Cancer *6*, 35. https://doi.org/10.1186/s40425-018-0342-x.
11. FDA approves pembrolizumab for adults and children with TMB-H solid tumors. https://www.fda.gov/drugs/drug-approvals-and-databases/fda-approves-pembrolizumab-adults-and-children-tmb-h-solid-tumors.
12. Overman, M.J., McDermott, R., Leach, J.L., Lonardi, S., Lenz, H.J., Morse, M.A., Desai, J., Hill, A., Axelson, M., Moss, R.A., et al. (2017). Nivolumab in patients with metastatic DNA mismatch repair-deficient or microsatellite instability-high colorectal cancer (CheckMate 142): an open-label, multicentre, phase 2 study. Lancet Oncol. *18*, 1182–1191. https://doi.org/10.1016/S1470-2045(17)30422-9.
13. Marcus, L., Lemery, S.J., Keegan, P., and Pazdur, R. (2019). FDA Approval Summary: Pembrolizumab for the Treatment of Microsatellite Instability-High Solid Tumors. Clin. Cancer Res. *25*, 3753–3758. https://doi.org/10.1158/1078-0432.CCR-18-4070.
14. Chen, P.L., Roh, W., Reuben, A., Cooper, Z.A., Spencer, C.N., Prieto, P.A., Miller, J.P., Bassett, R.L., Gopalakrishnan, V., Wani, K., et al. (2016). Analysis of Immune Signatures in Longitudinal Tumor Samples Yields Insight into Biomarkers of Response and Mechanisms of Resistance to Immune Checkpoint Blockade. Cancer Discov. *6*, 827–837. https://doi.org/10.1158/2159-8290.CD-15-1545.
15. Ayers, M., Lunceford, J., Nebozhyn, M., Murphy, E., Loboda, A., Kaufman, D.R., Albright, A., Cheng, J.D., Kang, S.P., Shankaran, V., et al. (2017). IFN-gamma-related mRNA profile predicts clinical response to PD-1 blockade. J. Clin. Investig. *127*, 2930–2940. https://doi.org/10.1172/JCI91190.
16. Jiang, P., Gu, S., Pan, D., Fu, J., Sahu, A., Hu, X., Li, Z., Traugh, N., Bu, X., Li, B., et al. (2018). Signatures of T cell dysfunction and exclusion predict cancer immunotherapy response. Nat. Med. *24*, 1550–1558. https://doi.org/10.1038/s41591-018-0136-1.

CellPress
OPEN ACCESS

17. Wang, H., Li, S., Wang, Q., Jin, Z., Shao, W., Gao, Y., Li, L., Lin, K., Zhu, L., Wang, H., et al. (2021). Tumor immunological phenotype signature-based high-throughput screening for the discovery of combination immunotherapy compounds. Sci. Adv. *7*, eabd7851. https://doi.org/10.1126/sciadv.abd7851.

18. Kiran, K.L., and Lakshminarayanan, S. (2013). Optimization of chemotherapy and immunotherapy: in silico analysis using pharmacokinetic–pharmacodynamic and tumor growth models. J. Process Control *23*, 396–403.

19. d'Onofrio, A., Ledzewicz, U., and Schättler, H. (2012). On the dynamics of tumor-immune system interactions and combined chemo-and immunotherapy. In New challenges for cancer systems biomedicine (Springer), pp. 249–266.

20. de Pillis, L., Fister, K.R., Gu, W., Collins, C., Daub, M., Gross, D., Moore, J., and Preskill, B. (2009). Mathematical model creation for cancer chemo-immunotherapy. Comput. Math. Methods Med. *10*, 165–184.

21. Lesterhuis, W.J., Rinaldi, C., Jones, A., Rozali, E.N., Dick, I.M., Khong, A., Boon, L., Robinson, B.W., Nowak, A.K., Bosco, A., and Lake, R.A. (2015). Network analysis of immunotherapy-induced regressing tumours identifies novel synergistic drug combinations. Sci. Rep. *5*, 12298. https://doi.org/10.1038/srep12298.

22. Xia, Y., Li, X., Bie, N., Pan, W., Miao, Y.R., Yang, M., Gao, Y., Chen, C., Liu, H., Gan, L., and Guo, A.Y. (2024). A method for predicting drugs that can boost the efficacy of immune checkpoint blockade. Nat. Immunol. *25*, 659–670. https://doi.org/10.1038/s41590-024-01789-x.

23. Li, Y., Burgman, B., McGrail, D.J., Sun, M., Qi, D., Shukla, S.A., Wu, E., Capasso, A., Lin, S.Y., Wu, C.J., et al. (2020). Integrated Genomic Characterization of the Human Immunome in Cancer. Cancer Res. *80*, 4854–4867. https://doi.org/10.1158/0008-5472.CAN-20-0384.

24. Jerby-Arnon, L., Shah, P., Cuoco, M.S., Rodman, C., Su, M.J., Melms, J. C., Leeson, R., Kanodia, A., Mei, S., Lin, J.R., et al. (2018). A Cancer Cell Program Promotes T Cell Exclusion and Resistance to Checkpoint Blockade. Cell *175*, 984–997.e24.

25. Gu, S.S., Zhang, W., Wang, X., Jiang, P., Traugh, N., Li, Z., Meyer, C., Stewig, B., Xie, Y., Bu, X., et al. (2021). Therapeutically Increasing MHC-I Expression Potentiates Immune Checkpoint Blockade. Cancer Discov. *11*, 1524–1541. https://doi.org/10.1158/2159-8290.CD-20-0812.

26. Ding, L., Kim, H.J., Wang, Q., Kearns, M., Jiang, T., Ohlson, C.E., Li, B.B., Xie, S., Liu, J.F., Stover, E.H., et al. (2018). PARP Inhibition Elicits STING-Dependent Antitumor Immunity in Brca1-Deficient Ovarian Cancer. Cell Rep. *25*, 2972–2980.e5. https://doi.org/10.1016/j.celrep.2018.11.054.

27. Sun, Y., Revach, O.Y., Anderson, S., Kessler, E.A., Wolfe, C.H., Jenney, A., Mills, C.E., Robitschek, E.J., Davis, T.G.R., Kim, S., et al. (2023). Targeting TBK1 to overcome resistance to cancer immunotherapy. Nature *615*, 158–167. https://doi.org/10.1038/s41586-023-05704-6.

28. Jenkins, R.W., Aref, A.R., Lizotte, P.H., Ivanova, E., Stinson, S., Zhou, C. W., Bowden, M., Deng, J., Liu, H., Miao, D., et al. (2018). Ex Vivo Profiling of PD-1 Blockade Using Organotypic Tumor Spheroids. Cancer Discov. *8*, 196–215. https://doi.org/10.1158/2159-8290.CD-17-0833.

29. Information, N.C.f.B. (2025). PubChem Compound Summary for CID 644215, Cgp-60474. https://pubchem.ncbi.nlm.nih.gov/compound/Cgp-60474.

30. Huang, W., Hickson, L.J., Eirin, A., Kirkland, J.L., and Lerman, L.O. (2022). Cellular senescence: the good, the bad and the unknown. Nat. Rev. Nephrol. *18*, 611–627. https://doi.org/10.1038/s41581-022-00601-z.

31. Ou, D.L., Chen, C.W., Hsu, C.L., Chung, C.H., Feng, Z.R., Lee, B.S., Cheng, A.L., Yang, M.H., and Hsu, C. (2021). Regorafenib enhances antitumor immunity via inhibition of p38 kinase/Creb1/Klf4 axis in tumor-associated macrophages. J. Immunother. Cancer *9*, e001657. https://doi.org/10.1136/jitc-2020-001657.

32. Fujimura, T., and Aiba, S. (2020). Significance of Immunosuppressive Cells as a Target for Immunotherapies in Melanoma and Non-Melanoma Skin Cancers. Biomolecules *10*, 1087. https://doi.org/10.3390/biom10081087.

33. Auslander, N., Zhang, G., Lee, J.S., Frederick, D.T., Miao, B., Moll, T., Tian, T., Wei, Z., Madan, S., Sullivan, R.J., et al. (2018). Robust prediction of response to immune checkpoint blockade therapy in metastatic melanoma. Nat. Med. *24*, 1545–1549. https://doi.org/10.1038/s41591-018-0157-9.

34. Riaz, N., Havel, J.J., Makarov, V., Desrichard, A., Urba, W.J., Sims, J.S., Hodi, F.S., Martín-Algarra, S., Mandal, R., Sharfman, W.H., et al. (2017). Tumor and Microenvironment Evolution during Immunotherapy with Nivolumab. Cell *171*, 934–949.e16. https://doi.org/10.1016/j.cell.2017.09.028.

35. Gide, T.N., Quek, C., Menzies, A.M., Tasker, A.T., Shang, P., Holst, J., Madore, J., Lim, S.Y., Velickovic, R., Wongchenko, M., et al. (2019). Distinct Immune Cell Populations Define Response to Anti-PD-1 Monotherapy and Anti-PD-1/Anti-CTLA-4 Combined Therapy. Cancer Cell *35*, 238–255.e6. https://doi.org/10.1016/j.ccell.2019.01.003.

36. Nathanson, T., Ahuja, A., Rubinsteyn, A., Aksoy, B.A., Hellmann, M.D., Miao, D., Van Allen, E., Merghoub, T., Wolchok, J.D., Snyder, A., and Hammerbacher, J. (2017). Somatic Mutations and Neoepitope Homology in Melanomas Treated with CTLA-4 Blockade. Cancer Immunol. Res. *5*, 84–91. https://doi.org/10.1158/2326-6066.CIR-16-0019.

37. Amato, C.M., Hintzsche, J.D., Wells, K., Applegate, A., Gorden, N.T., Vorwald, V.M., Tobin, R.P., Nassar, K., Shellman, Y.G., Kim, J., et al. (2020). Pre-Treatment Mutational and Transcriptomic Landscape of Responding Metastatic Melanoma Patients to Anti-PD1 Immunotherapy. Cancers (Basel) *12*, 1943. https://doi.org/10.3390/cancers12071943.

38. Miao, D., Margolis, C.A., Gao, W., Voss, M.H., Li, W., Martini, D.J., Norton, C., Bossé, D., Wankowicz, S.M., Cullen, D., et al. (2018). Genomic correlates of response to immune checkpoint therapies in clear cell renal cell carcinoma. Science *359*, 801–806. https://doi.org/10.1126/science.aan5951.

39. Braun, D.A., Hou, Y., Bakouny, Z., Ficial, M., Sant' Angelo, M., Forman, J., Ross-Macdonald, P., Berger, A.C., Jegede, O.A., Elagina, L., et al. (2020). Interplay of somatic alterations and immune infiltration modulates response to PD-1 blockade in advanced clear cell renal cell carcinoma. Nat. Med. *26*, 909–918. https://doi.org/10.1038/s41591-020-0839-y.

40. Kim, J.Y., Choi, J.K., and Jung, H. (2020). Genome-wide methylation patterns predict clinical benefit of immunotherapy in lung cancer. Clin. Epigenetics *12*, 119. https://doi.org/10.1186/s13148-020-00907-4.

41. Cho, J.W., Hong, M.H., Ha, S.J., Kim, Y.J., Cho, B.C., Lee, I., and Kim, H. R. (2020). Genome-wide identification of differentially methylated promoters and enhancers associated with response to anti-PD-1 therapy in non-small cell lung cancer. Exp. Mol. Med. *52*, 1550–1563. https://doi.org/10.1038/s12276-020-00493-8.

42. Kim, S.T., Cristescu, R., Bass, A.J., Kim, K.M., Odegaard, J.I., Kim, K., Liu, X.Q., Sher, X., Jung, H., Lee, M., et al. (2018). Comprehensive molecular characterization of clinical responses to PD-1 inhibition in metastatic gastric cancer. Nat. Med. *24*, 1449–1458. https://doi.org/10.1038/s41591-018-0101-z.

43. Zhao, J., Chen, A.X., Gartrell, R.D., Silverman, A.M., Aparicio, L., Chu, T., Bordbar, D., Shan, D., Samanamud, J., Mahajan, A., et al. (2019). Immune and genomic correlates of response to anti-PD-1 immunotherapy in glioblastoma. Nat. Med. *25*, 462–469. https://doi.org/10.1038/s41591-019-0349-y.

44. Mariathasan, S., Turley, S.J., Nickles, D., Castiglioni, A., Yuen, K., Wang, Y., Kadel, E.E., III, Koeppen, H., Astarita, J.L., Cubas, R., et al. (2018). TGFbeta attenuates tumour response to PD-L1 blockade by contributing to exclusion of T cells. Nature *554*, 544–548. https://doi.org/10.1038/nature25501.

45. Liu, D., Schilling, B., Liu, D., Sucker, A., Livingstone, E., Jerby-Arnon, L., Zimmer, L., Gutzmer, R., Satzger, I., Loquai, C., et al. (2019). Integrative molecular and clinical modeling of clinical outcomes to PD1 blockade in patients with metastatic melanoma. Nat. Med. *25*, 1916–1927. https://doi.org/10.1038/s41591-019-0654-5.

46. Garcia-Diaz, A., Shin, D.S., Moreno, B.H., Saco, J., Escuin-Ordinas, H., Rodriguez, G.A., Zaretsky, J.M., Sun, L., Hugo, W., Wang, X., et al.

(2017). Interferon Receptor Signaling Pathways Regulating PD-L1 and PD-L2 Expression. Cell Rep. *19*, 1189–1201. https://doi.org/10.1016/j.celrep.2017.04.031.

47. Lauss, M., Donia, M., Harbst, K., Andersen, R., Mitra, S., Rosengren, F., Salim, M., Vallon-Christersson, J., Törngren, T., Kvist, A., et al. (2017). Mutational and putative neoantigen load predict clinical benefit of adoptive T cell therapy in melanoma. Nat. Commun. *8*, 1738. https://doi.org/10.1038/s41467-017-01460-0.
48. Rose, T.L., Weir, W.H., Mayhew, G.M., Shibata, Y., Eulitt, P., Uronis, J.M., Zhou, M., Nielsen, M., Smith, A.B., Woods, M., et al. (2021). Fibroblast growth factor receptor 3 alterations and response to immune checkpoint inhibition in metastatic urothelial cancer: a real world experience. Br. J. Cancer *125*, 1251–1260. https://doi.org/10.1038/s41416-021-01488-6.
49. Colaprico, A., Silva, T.C., Olsen, C., Garofano, L., Cava, C., Garolini, D., Sabedot, T.S., Malta, T.M., Pagnotta, S.M., Castiglioni, I., et al. (2016). TCGAbiolinks: an R/Bioconductor package for integrative analysis of TCGA data. Nucleic Acids Res. *44*, e71. https://doi.org/10.1093/nar/gkv1507.
50. Ghoshdastider, U., Rohatgi, N., Mojtabavi Naeini, M., Baruah, P., Revkov, E., Guo, Y.A., Rizzetto, S., Wong, A.M.L., Solai, S., Nguyen, T.T., et al. (2021). Pan-Cancer Analysis of Ligand-Receptor Cross-talk in the Tumor Microenvironment. Cancer Res. *81*, 1802–1812. https://doi.org/10.1158/0008-5472.CAN-20-2352.
51. Aran, D., Sirota, M., and Butte, A.J. (2015). Systematic pan-cancer analysis of tumour purity. Nat. Commun. *6*, 8971. https://doi.org/10.1038/ncomms9971.
52. Yu, G., Wang, L.G., Han, Y., and He, Q.Y. (2012). clusterProfiler: an R package for comparing biological themes among gene clusters. OMICS *16*, 284–287. https://doi.org/10.1089/omi.2011.0118.
53. Subramanian, A., Narayan, R., Corsello, S.M., Peck, D.D., Natoli, T.E., Lu, X., Gould, J., Davis, J.F., Tubelli, A.A., Asiedu, J.K., et al. (2017). A Next Generation Connectivity Map: L1000 Platform and the First 1,000,000 Profiles. Cell *171*, 1437–1452.e17. https://doi.org/10.1016/j.cell.2017.10.049.
54. Liu, Y., Chen, S., Li, Z., Morrison, A.C., Boerwinkle, E., and Lin, X. (2019). ACAT: A Fast and Powerful p Value Combination Method for Rare-Variant Analysis in Sequencing Studies. Am. J. Hum. Genet. *104*, 410–421. https://doi.org/10.1016/j.ajhg.2019.01.002.
55. Chen, B., Ma, L., Paik, H., Sirota, M., Wei, W., Chua, M.S., So, S., and Butte, A.J. (2017). Reversal of cancer gene expression correlates with drug efficacy and reveals therapeutic targets. Nat. Commun. *8*, 16022. https://doi.org/10.1038/ncomms16022.
56. Yu, K., Chen, B., Aran, D., Charalel, J., Yau, C., Wolf, D.M., van 't Veer, L.J., Butte, A.J., Goldstein, T., and Sirota, M. (2019). Comprehensive transcriptomic analysis of cell lines as models of primary tumors across 22 tumor types. Nat. Commun. *10*, 3574. https://doi.org/10.1038/s41467-019-11415-2.
57. Wishart, D.S., Feunang, Y.D., Guo, A.C., Lo, E.J., Marcu, A., Grant, J.R., Sajed, T., Johnson, D., Li, C., Sayeeda, Z., et al. (2018). DrugBank 5.0: a major update to the DrugBank database for 2018. Nucleic Acids Res. *46*, D1074–D1082. https://doi.org/10.1093/nar/gkx1037.
58. Zhou, Y., Zhang, Y., Lian, X., Li, F., Wang, C., Zhu, F., Qiu, Y., and Chen, Y. (2022). Therapeutic target database update 2022: facilitating drug discovery with enriched comparative data of targeted agents. Nucleic Acids Res. *50*, D1398–D1407. https://doi.org/10.1093/nar/gkab953.
59. Glassman, C.R., Mathiharan, Y.K., Jude, K.M., Su, L., Panova, O., Lupardus, P.J., Spangler, J.B., Ely, L.K., Thomas, C., Skiniotis, G., and Garcia, K.C. (2021). Structural basis for IL-12 and IL-23 receptor sharing reveals a gateway for shaping actions on T versus NK cells. Cell *184*, 983–999.e24. https://doi.org/10.1016/j.cell.2021.01.018.
60. Tang, F., Zhao, L.T., Jiang, Y., Ba, D.N., Cui, L.X., and He, W. (2008). Activity of recombinant human interleukin-15 against tumor recurrence and metastasis in mice. Cell. Mol. Immunol. *5*, 189–196. https://doi.org/10.1038/cmi.2008.23.
61. Humphrey, S.J., Azimifar, S.B., and Mann, M. (2015). High-throughput phosphoproteomics reveals in vivo insulin signaling dynamics. Nat. Biotechnol. *33*, 990–995. https://doi.org/10.1038/nbt.3327.
62. Wang, L., Bi, S., Li, Z., Liao, A., Li, Y., Yang, L., Zhou, X., Gao, Y., Liu, X., Zou, Y., et al. (2025). Napabucasin deactivates STAT3 and promotes mitoxantrone-mediated cGAS-STING activation for hepatocellular carcinoma chemo-immunotherapy. Biomaterials *313*, 122766. https://doi.org/10.1016/j.biomaterials.2024.122766.
63. Wang, Z., Su, Q., Deng, W., Wang, X., Zhou, H., Zhang, M., Lin, W., Xiao, J., and Duan, X. (2024). Morphology-Mediated Tumor Deep Penetration for Enhanced Near Infrared II Photothermal and Chemotherapy of Colorectal Cancer. ACS Nano *18*, 28038–28051. https://doi.org/10.1021/acsnano.4c07085.
64. Tian, F., Conde, J., Bao, C., Chen, Y., Curtin, J., and Cui, D. (2016). Gold nanostars for efficient in vitro and in vivo real-time SERS detection and drug delivery via plasmonic-tunable Raman/FTIR imaging. Biomaterials *106*, 87–97. https://doi.org/10.1016/j.biomaterials.2016.08.014.

STAR★METHODS

KEY RESOURCES TABLE

REAGENT or RESOURCE	SOURCE	IDENTIFIER
Antibodies		
anti-CD45	Abcam	Cat# ab40763; RRID: AB_726545
anti-CD3	Abcam	Cat# ab11089; RRID: AB_2889189
anti-CD8	Cell Signaling Technology	Cat# 98941; RRID: AB_2756376
anti-PD-1	Abcam	Cat# ab237728; RRID: AB_3073606
anti-PDL-1	Abcam	Cat# ab237726; RRID: AB_2884992
Bacterial and virus strains		
pT3-EF1a-*c*-myc	Addgene	Cat# 92046; RRID: Addgene_92046
pX330 p53	Addgene	Cat# 59910; RRID: Addgene; #59910
Sleeping Beauty SB100x transposase-encoding plasmid	Addgene	Cat# 34879; RRID: Addgene_34879
Biological samples		
Human tumor tissues from cancer patients	Participants in this study	N/A
Chemicals, peptides, and recombinant proteins		
anti-PD-1 pembrolizumab	MCE	Cat# HY-P9902A; RRID: AB_3694335
Docetaxel	MCE	Cat# HY-B0011
Trametinnib	MCE	Cat# HY-10999
PD-0325901	MCE	Cat# HY-10254
Tubacin	MCE	Cat# HY-13428
Tyrphostin-AG-494	MCE	Cat# HY-101042
SB-366791	MCE	Cat# HY-12245
Phenothiazine	MCE	Cat# HY-Y0055
CGP-60474	MCE	Cat# HY-11009
anti–PD-1 monoclonal antibody	Bio X Cell	Cat# BE0146; RRID: AB_10949053
isotype control monoclonal antibody	Bio X Cell	Cat# BE0089; RRID: AB_1107769
Mitoxantrone	MCE	Cat# HY-13502
Matrigel Matrix	Corning	Cat# 356231
DMEM	Gibco	Cat# 1965092
FBS	Gibco	Cat# 10099141C
penicillin/streptomycin	Gibco	Cat# 15140122
Advanced DMEM/F-12	Thermo Fisher Scientific	Cat# 12634010
collagenase, Type 4	Worthington	Cat# LS004188
hyaluronidase	Sigma	Cat# H3506
deoxyribonuclease	Sigma	Cat# DN25
RBC lysis buffer	Beyotime	Cat# C3702
Nexcelom ViaStain AO/PI Staining Solution	Nexcelom	Cat# CS2-0106
Critical commercial assays		
RNA bulk sequencing	Majorbio	N/A
Cell Counting Kit-8	Meilunbio	Cat# MA0218
High-content screening	PerkinElmer	Cat# Operetta CLS
Deposited data		
Transcriptomes and phenotypes of human and mouse immunotherapy cohorts	Previous papers	Zenodo: https://doi.org/10.5281/zenodo.15259448

(*Continued on next page*)

Continued

REAGENT or RESOURCE	SOURCE	IDENTIFIER
RNA bulk sequencing from liver cancer tissues of mice	This paper	NODE: https://www.biosino.org/node/project/detail/OEP00006029
**Experimental models: Cell lines**		
MC38	Ubigene	Cat# YC-A002; RRID: CVCL_B288
LA795	Pricella	Cat# CL-0376
Huh7	NICR	Cat# 1101HUM-PUMC000679
Hep3B	ATCC	Cat# HB-8064; RRID: CVCL_0326
Hepa1-6	ATCC	Cat# CRL-1830; RRID: CVCL_0327
**Experimental models: Organisms/strains**		
C57BL/6J mice	Shanghai SLAC Laboratory Animal Co., Ltd.	N/A
BALB/c nude mice	Shanghai SLAC Laboratory Animal Co., Ltd.	N/A
T739 mice	Shanghai SLAC Laboratory Animal Co., Ltd.	N/A
**Software and algorithms**		
Code of IGeS-BS	This paper	https://doi.org/10.5281/zenodo.15787405
GSEA version 3.0	https://www.gsea-msigdb.org/gsea/index.jsp	N/A
R version 4.0.2	https://www.r-project.org	N/A
TCGAbiolinks version 2.16.0	Bioconductor	N/A
TIDE (webserver)	http://tide.dfci.harvard.edu/l	N/A
Immunedeconv version 2.0.2	https://github.com/omnideconv/immunedeconv.git	N/A
MCPcounter version 1.1.0	https://github.com/ebecht/MCPcounter.git	N/A
Immucellai (webserver)	https://guolab.wchscu.cn/ImmuCellAI//#!/	N/A
xCell	https://github.com/dviraran/xCell.git	N/A
ConsensusTME	https://github.com/cansysbio/ConsensusTME	N/A
Immunophenogram	https://github.com/icbi-lab/Immunophenogram.git	N/A
Immune-Subtype-Clustering	https://github.com/Gibbsdavidl/Immune-Subtype-Clustering.git	N/A
ImmuneResistance	https://github.com/livnatje/ImmuneResistance.git	N/A
CIBERSORT	https://cibersortx.stanford.edu/	N/A
ACAT (Aggregated Cauchy Assocaition Test)	https://github.com/yaowuliu/ACAT.git	N/A
clusterProfiler version 3.18.1	https://guangchuangyu.github.io/software/clusterProfiler	N/A
GraphPad PRISM version 8.0.2	https://www.graphpad.com/updates/prism-802-release-notes	N/A

EXPERIMENTAL MODEL AND STUDY PARTICIPANT DETAILS

Animal studies

All animal care and experimental protocols were approved by the Institutional Animal Care and Use Committee (IACUC) of Zhongshan Hospital, Fudan University (Approval Number 20220211-21). Six-week-old male C57BL/6J mice, BALB/c nude mice and T739 mice were purchased from Shanghai SLAC Laboratory Animal Co., Ltd., and all the animals used in the study were fed in a specific pathogen-free facility. C57BL/6J and T739 mice are immunocompetent while BALB/c nude mice is immunodeficient.

Patient material

Tumor samples were obtained from patients post-surgery. The study protocol was approved by the Ethics Committee of Zhongshan Hospital (Approval Number B2022-063R, B2025-159R) and was in accordance with the Declaration of Helsinki. In experiments of

8 compounds, HCC Patient 1 is Male, 88 years old; HCC Patient 2 is Male, 53 years old; HCC Patient 3 is Female, 78 years old. In experiments of mitoxantrone, HCC Patient 1 is Male, 77 years old; HCC Patient 2 is Male, 71 years old; HCC Patient 1 is Male, 69 years old. In experiments of SB-366791 and mitoxantrone, colon cancer patient 1 is Male, 67 years old; colon cancer patient 2 is Male, 80 years old; colon cancer patient 3 is Female, 57 years old; lung adenocarcinoma patient 1 is Male, 61 years old; lung adenocarcinoma patient 1 is Male, 59 years old; lung adenocarcinoma patient 3 is Female, 49 years old. The health status of patients was assessed with Eastern Cooperative Oncology Group performance status (ECOG PS), all patients had ECOG PS $\leq$ 1 (10 patients were PS = 0 and 2 patients were PS = 1). All patients were immunocompetent. All patients didn't receive previous treatment before surgery. Written informed consent was obtained from all patients prior to involvement in the study.

METHOD DETAILS

Immunotherapy pancancer cohorts

To collect datasets of human immune checkpoint blockade therapy, we designated December 2021 as the cutoff point and retrieved 44 datasets from the PubMed and GEO databases by using keywords "PD1", "PD-L1", "anti PD1", "anti-PDL1", "immunotherapy" and "immune checkpoint inhibitor". Among them, seventeen cohorts with transcriptomic data before immunotherapy and response information were collected, and randomly split into discovery and validation sets without any artificial bias regarding the prediction accuracy (Table S1). The criteria for selecting the discovery set are either having a larger sample size or being the sole dataset accessible for a particular cancer type. The discovery sets include 12 cohorts of 6 cancer types: five melanoma datasets,[33–37] two clear cell renal cell carcinoma datasets,[38,39] two lung cancer datasets,[40,41] one stomach cancer dataset,[42] one glioblastoma dataset[43] and one bladder cancer dataset.[41,44] The validation set has 5 cohorts of 2 cancer types: four melanoma datasets[33,45–47] and one bladder cancer dataset.[48] Treatment response evaluation and transcriptomic data processing for all cohorts were performed with a similar pipeline.

TCGA pancancer cohorts

Twenty-one kinds of solid tumors from the TCGA database were used, including primary bladder urothelial carcinoma (BLCA), BRCA, cervical squamous cell carcinoma and endocervical adenocarcinoma (CESC), COAD, esophageal carcinoma (ESCA), glioblastoma multiforme (GBM), head and neck squamous cell carcinoma (HNSC), kidney renal clear cell carcinoma (KIRC), kidney renal papillary cell carcinoma (KIRP), low grade glioma (LGG), LIHC, lung adenocarcinoma (LUAD), lung squamous cell carcinoma (LUSC), ovarian serous cystadenocarcinoma (OV), pancreatic adenocarcinoma (PAAD), PRAD, READ, stomach adenocarcinoma (STAD), thyroid carcinoma (THCA), uterine corpus endometrial carcinoma (UCEC), primary SKCM (pSKCM) and metastatic SKCM (mSKCM). RNA sequencing data and clinical information were downloaded via TCGAbiolinks.[49] The gene expression matrix was converted to TPM values. We downloaded the consensus tumor purity estimated with the TUMERIC method from.[50] Primary SKCM was not provided by the TUMERIC method; here, we used the tumor purity estimated by the CPE method.[51] Patients without estimated tumor purity data were excluded from the analysis.

Identification of immunotherapy-related gene expression signatures

Three categories of TME-related gene sets (TME_GS) were manually curated from publications, and their activity scores in each tumor sample were calculated based on the previous described methods (Table S1). For each TME_GS, we analyzed its association with immunotherapy response via the discovery sets. In each cohort, the original scores of each gene set were standardized via Z-transformation, with a mean of 0 and a standard deviation of 1. A logistic regression model was established via the R function "glm" to estimate the effect size (regression coefficient) and residuals for each TME_GS, where the standardized score and the binarized response label were taken as the independent variable and dependent variable, respectively. Considering the heterogeneity among cohorts, a random-effects model was applied for meta-analysis via the R package "meta" to integrate the effect sizes of each gene set in the discovery cohort, and the odds ratio (OR) and *p* value indicating the association between gene sets and immunotherapy response were obtained. Ultimately, TME_GS signatures with *p* values less than 0.05 and ORs greater than 1 were defined as immunotherapy sensitivity-related signatures (sIGeS), and those with *p* values less than 0.05 and OR ranges of 0–1 were defined as immunotherapy resistance-related signatures (rIGeS).

Owing to the differences among different cancer types and the changes in the subsequent quantification of IGeS using compound-induced cancer cell lines, an expansion strategy was used to make IGeS detectable in diverse cancer types and cell lines. For each cancer type in TCGA, tumor purity was taken as a confounding variable, and the partial Spearman correlation coefficient (PSCC) and adjusted *p*-value (FDR) between each IGeS and each gene was calculated via the R package "ppcor". The detect ability of genes within CCLE cancer cell lines was further defined as the proportion of cancer cell lines in which at least one read were detect for this gene. Genes with PSCC >0.4, FDR <0.05 and DP > 0.8 were selected as the cancer type-specific IGeS; if gene set is too large, genes are retricted to top 200 genes. IGeSs were further renamed based on the original signatures and functional enrichment on Rectome pathway terms with R package ClusterProfile.[52]

Construction and evaluation of the immunotherapy response prediction model

To evaluate the accuracy of IGeS combinations in predicting immunotherapy responses, five multivariable machine learning models were built, including elastic net regression (IGeS-EN), ridge regression (IGeS-RR), random forest (IGeS-RF), support vector machine

(IGeS-SVM) and the gradient boosted tree algorithm (IGeS-XGBoost). Before training, combat was applied to remove the batch effect between cohorts. Fivefold cross validation (CV) was applied to assess the performance on the discovery sets. For each fold, the model was trained on 4/5 of the data with an additional 10-fold CV for hyperparameter tuning and was tested on the remaining fold. Each test fold was layered together to generate the overall performance on the discovery sets. Additionally, the models were retrained on the whole discovery set in the same manner for hyperparameter optimization and were applied to the five independent validation sets to assess the performance and generalizability of the models. Elastic net regression, random forest, support vector machine and XGBoost were constructed under the "mlr" framework; the ridge regression model was trained via the "glmnet" package with the same data split strategy as others; and evaluation matrices (i.e., ROC curves and AUC values) were calculated via the "pROC" package in R.

Furthermore, our models were compared with published transcriptome-based immunotherapy prediction methods, including the mean expression of IFNG signature genes,[16] the mean expression of CD8 signature genes,[16] the 18-gene score,[15,16] the TIP score,[17] the Immunophenoscore (IPS),[8] the T-cell exclusion program (T-exclu)[24] and the TIDE model.[16] These methods were calculated locally via the codes provided by the original literature or rewritten scripts according to the descriptions from the original publications.

Compound-induced gene expression datasets

The compound-induced gene expression profiles of the cancer cell lines were downloaded from the LINCS project,[53] which provides two batches of data (GEO: GSE70138 and GSE92742). The level 3 LINCS data were parsed by the parse.gctx() function to obtain the gene expression profiles of each compound under different doses and treatment times as well as dimethyl sulfoxide (DMSO)-treated profiles on each plate as the control group. The tissue origins and cancer types of the cancer cell lines in the LINCS datasets and patients in the TCGA dataset were matched via the same nomenclature. After the matching criteria were met, 13 cancer types with matching drug-perturbed gene expression profiles in cancer cell lines were retained for subsequent analysis. GEO: GSE70138 contains 1768 compounds, 12 cell lines from 8 cancer types (BRCA, LIHC, LUAD, PAAD, PRAD, COAD, READ, SKCM), 202133 compounds-treated expression profiles, and 14304 DMSO-treated cells. GEO: GSE92742 contains 18486 compounds, 38 cell lines from 11 cancer types (BRCA, COAD, LIHC, LUAD, LUSC, OV, PRAD, READ, SKCM, STAD, and UCEC), 443505 compounds-treated expression profiles, and 23252 DMSO-treated expression profiles. Melanoma cell lines were used for both primary SKCM (pSKCM) and metastatic SKCM (mSKCM). Colorectal cancer cell lines were used for both primary COAD and READ.

Quantification of the effects of compounds on IGeS expression

The LINCS dataset contains multiple cell lines for each cancer type and multiple perturbation conditions (cell line, drug dose, treatment time) for a compound. For each perturbation condition (cell, dose, time), genes were ranked according to the expression difference between the drug-treated cell lines and the control group. Then, prerank GSEA was applied to calculate an NES score and *p* value, which reflects the influence of the compound on a given IGeS under a certain condition. To integrate the results from multiple perturbation conditions for each trio (IGeS, cancer type, compound), FDR-adjusted *p* values were combined via an aggregated Cauchy association test (ACAT),[54] and NES scores (normalized enrichment scores) were aggregated to obtain a more robust perturb gene expression score (PGES) via a published method[55] with slight modifications to include the scenario where only one dose and one time point of a compound were given.

$$\mathrm{PGES} = \begin{cases} \frac{1}{n}\sum_{p=1}^{n} W_{cell_p} * NES_{reference_p}, \textit{if only one dose and time point is tested} \\ \frac{1}{n}\sum_{p=1}^{n} W_{cell_p} * \left(NES_{reference_p} + f(dose_p, time_p)\right), \textit{if multiple doses and/or time points is tested}, \end{cases}$$

where n represents the number of perturbation conditions (cell, dose, time) for a given compound and cancer type and where $cell_p$ and $dose_p$ and $time_p$ represent the cell line, drug dose and treatment time, respectively, for the condition p. W_{cell_p} is the mean correlation coefficient between $cell_p$ and the corresponding patient samples in the same cancer type in TCGA divided by their maximum value.[56] If W_{cell_p} was not provided in,[56] the mean correlation for all the cell lines in the corresponding cancer type was used. The available doses and times are variable among compounds. Most compounds were assessed at 10 μM for 24 h and were regarded as references ($reference_p$) for other doses and times. $f(dose_p, time_p)$ is a straightforward awarding function to simplify the difference introduced by multiple (dose, time) combinations. The treatment doses and times are divided into (low, high) and (short, long) relative to the reference condition, and the average PGES under the condition (dose, time) that falls inside a certain basket is taken as the awarding value: $f(dose_p, time_p) = \frac{\sum_{condition=1}^{m}(PGES_{condition} - PGES_{reference_p})}{m}$, where m refers to the number of conditions (dose, time) in the corresponding condition.

In silico ranking of compounds for increasing immunotherapeutic response

We hypothesized that if some compounds could induce increased/decreased expression of sIGeS/rIGeS, such compounds could increase immunotherapy efficacy. On the basis of this hypothesis and the IGeS-EN model, a boosting score (BS) was defined to integrate the expression changes of all IGeSs while controlling the multicollinearity among IGeSs:

$$BS = \sum_{i \in N} Coe_i * PGES_i$$

where i is the index of IGeS, N is the subset of IGeS with significant expression changes under compound perturbation (FDR adjusted *p* value < 0.05), $PGES_i$ is the summed "perturb gene expression score" for the trio ($IGeS_i$, cancer type, compound), and Coe_i is the regression coefficient of $IGeS_i$ extracted from the IGeS-EN model. A higher BS value reflects the potential of a drug or compound to increase immunotherapy efficacy for a given cancer type.

Evaluation of the IGeS-BS with prior data and knowledge

The annotations of drugs or compounds, including the clinical phase, mechanism of action and target genes, were downloaded and integrated from the API of lincs_portal2.0 (lincsportal.ccs.miami.edu), DrugBank v5.1[57] and the Therapeutic Target Database (TTD).[58]

First, we investigated whether compound-induced cell lines could reflect *in vivo* changes by comparing expression changes estimated from LINCS and those from cancer mouse models. We collected five expression datasets of immunocompetent mice before and after ICB single or combination therapy: GEO: GSE149825, GSE152925, GSE120500, GSE114601, and GSE160785 (Table S6). The log-transformed expression values (normalized counts or FPKMs) were used to calculate the fold changes before and after treatment. The genes ranked by fold changes were subjected to GSEA to analyse the enrichment of IGeS genes. IGeS with p.adj <0.1 were considered to be regulated after drug treatment. The consistency between cancer cell lines and animal models under the same drug treatment was assessed by counting the proportion of IGeS with the same regulatory direction.

Second, we compared the predicted results of IGeS-BS with prior knowledge. ICB combinations approved by the FDA or in clinical trials were manually curated from https://www.fda.gov and https://clinicaltrials.gov/as supporting evidence. The effect size is defined as the difference in mean scores between the compound with and without supporting evidence. The single-sided Wilcoxon rank-sum test was applied to examine whether the scores for the compounds were significantly higher in the group with supporting evidence.

Experimental validation of IGeS-BS-predicted compounds for liver cancer

Drug IC50 assay

The Huh7 (NICR; #1101HUM-PUMC000679) cell line was purchased from the National Infrastructure of Cell Line Resource. The Hep3B (ATCC; # HB-8064) cell line was purchased from the American Type Culture Collection. All the cell lines used in the research were authenticated via short tandem repeat (STR) analysis. Cell lines were tested mycoplasma contamination free before experiments. The cells were cultured in DMEM containing 10% FBS (Gibco; #10099141C) and 100 U/L penicillin/streptomycin (Gibco; #15140122) at 37°C and 5% CO_2 in a cell incubator. The logarithmic phase cells were digested, centrifuged and counted to 5 × 104 cells/ml. The cells were inoculated into 96-well plates (100 μL per well) and cultured at 37°C and 5% CO_2 for 24 h. The growth state and density of the cells were observed under an inverted microscope. The cells in the well-growing state with a uniform distribution and density were taken for experiments. For Hep3B cells, CGP-60474 was diluted to different concentrations: 0, 10, 20, 30.50, and 100 nM; Docetaxel solution concentrations: 0, 0.5, 1, 2, 5, and 10 nM; PD-0325901 solution concentrations: 0.01.05, 0.10, 0.20, and 1.0 μM; Trametinnib solution concentrations: 0.0, 10.25, 0.50, 0.75, and 1.0 μM; and SB-366791, Phenothiazine, Tyrphostin-AG-494, and Tubacin Solution concentrations: 0, 1, 5, 10, 25, and 50 μM. For Huh7 cells, the GP-60474 solution concentrations were 0, 10.50, 100, 150, and 200 nM; the docetaxel solution concentrations were 0, 0.5, 1, 2, 5, and 10 nM; and the trametinnib, PD-0325901, SB-366791, phenothiazine, Tyrphostin-AG-494 and tubacin solution concentrations were 0, 1, 5, 10, 25, and 50 μM. Three double wells per concentration were seeded and cultured at 37°C and 5% CO_2 for 72 h. Medium supplemented with 10% CCK-8 solution (Meilunbio; #MA0218) was added in the form of 100 μL of fluid exchange per well, and the mixture was incubated for 2 h at 37°C and 5% CO_2. After incubation, the OD values were measured at 450 nm via an enzyme-linked immunosorbent assay. Cell viability (%) = (dosing group OD value-blank group OD value)/(control group OD value-blank group OD value) × 100%. The experimental data were processed with GraphPad PRISM 8.0.2 statistical software.

PDOTs culture and ex vivo drug test

Patient-derived organotypic tumor spheroids (PDOTs) were prepared and cultured as previously described.[27] Fresh colon cancer, lung adenocarcinoma or hepatocellular carcinoma tumor tissues removed by surgery were minced in a sterile 10 cm dish using a scalpel after being washed 3 times with PBS. Minced tumors were diluted in Advanced DMEM/F-12 (Thermo Fisher Scientific; #12634010) supplemented with 0.1% collagenase, Type 4 (Worthington; #LS004188), 0.05% hyaluronidase (Sigma; #H3506), and 0.01% deoxyribonuclease (Sigma; #DN25) and shaken on a horizontal platform for 30 min at 37°C. Red blood cells (RBCs) were removed via RBC lysis buffer (Beyotime; C3702). The cell suspensions were then pelleted and resuspended in fresh media and passed through 100 mm and 40 mm filters sequentially to obtain S1 (>100 μM), S2 (40–100 μM), and S3 (<40 μM) spheroid fractions. The S2 fraction was resuspended in Matrigel (Corning; #356231) at a concentration of 2.5 mg/mL. The spheroid-Matrigel mixture was injected into the microfluidic culture device. After incubation for 15 min at 37°C in sterile humidity chambers, The matrigel containing

PDOTS was cultured in media supplemented with or without the indicated treatments: untreated control; anti-PD-1 (250 μg/mL pembrolizumab); docetaxel (1.95 ± 0.10 nM); combined docetaxel + anti-PD-1; CGP-60474 (28.59 ± 4.08 nM); combined CGP-60474 + anti-PD-1; trametinnib (10 μM); combined trametinnib + anti-PD-1; PD-0325901 (10 μM); combined PD-0325901 + anti-PD-1; tubacin (14.81 ± 0.38 μM); combined tubacin + anti-PD-1; Tyrphostin-AG-494 (10 μM); combined Tyrphostin-AG-494 + anti-PD-1; SB-366791 (10 μM); combined SB-366791 + anti-PD-1; phenothiazine (10 μM); combined phenothiazine + anti-PD-1; mitoxantrone (10 μM); or combined mitoxantrone + anti-PD-1.

PDOTs live/dead analysis

Dual labeling was performed by replacing the culture medium with Nexcelom ViaStain AO/PI Staining Solution (Nexcelom, #CS2-0106). Following incubation with the dyes (15 min at room temperature in the dark), images were captured via high-content screening (HCS, PerkinElmer). Image capture and analysis were performed via the Harmony™ analysis system (PerkinElmer). Live and dead cell quantification was performed by measuring the total cell area of each dye, while green was used for live cells and red was used for dead cells. Cell area = green area/total area.

Animal studies

Six-week-old male C57BL/6J mice were purchased from Charles River (Shanghai, China), and all the animals used in the study were fed in a specific pathogen-free facility. All animal care and experimental protocols were approved by the Institutional Animal Care and Use Committee (IACUC) of Zhongshan Hospital, Fudan University. To establish spontaneous tumorigenesis HCC models, 20 μg of pT3-EF1a-c-myc (Addgene; #92046), 20 μg of pX330 p53 (Addgene; #59910) and 10 μg of Sleeping Beauty SB100x transposase-encoding plasmid (Addgene; #34879) per mouse dissolved in 2 mL of 0.9% NaCl solution were injected into C57BL/6J mice through tail vein injection within 1–3 s. Two weeks after injection, the mice bearing spontaneous tumors were randomized into the indicated groups. For medical therapy, C57BL/6J tumor–bearing mice were treated intraperitoneally with 10 mg/kg CGP-60474 (MedChemExpress; #HY-11009), 20 mg/kg SB-366791 (MedChemExpress; #HY-12245) and/or 10 mg/kg anti–PD-1 monoclonal antibody (Bio X Cell, #BE0146) or isotype control monoclonal antibody (Bio X Cell, #BE0089) following the schedule shown in Figure 6A. Briefly, drug therapy was administered every 2 days for a total of 7 times.

Proper cell lines were chosen for corresponding cancers. MC38[59] (colon cancer), LA795[60] (lung adenocarcinoma) and Hepa1-6[61] (HCC) cell lines were used to construct subcutaneous tumors. MC38 and Hepa1-6 were injected in C57BL/6J mouse, LA795 were injected in T739 mouse. SB-366791 and its combination therapy were applied in MC38 and LA795. Mitoxantrone and its combination therapy were applied in MC38, LA795 and Hepa1-6. The specific drug dosage is as follows: anti–PD-1 monoclonal antibody (10mg/kg), isotype control monoclonal antibody (10mg/kg), SB-366791 (20mg/kg), Mitoxantrone (1mg/kg in Hepa1-6,[62] 5mg/kg in MC38[63] and 10mg/kg in LA795[64]). Treatment was applied every 2 days, 7 times in total.

Immunofluorescence

Paraffin-embedded tissue samples were deparaffinized, followed by citrate buffer antigen retrieval at 95°C and blocking. Samples were then incubated with primary antibody at 4°C overnight and secondary antibody at 37°C for 30 min. Nuclei were stained with DAPI. Images were acquired using a THUNDER Imaging System (Leica, Germany).

RNAseq analysis

Tumor tissues from three mice of each condition were subjected to RNAseq, which generated the expression profiles of 18 samples under 6 conditions (Ctrl, PD1, CGP-60474, CGP-60474 and PD1, SB-366791, SB-366791 and PD1). Differential genes between the treatment and control groups were identified by DESeq2 and filtered by 'adjusted p value <0.1' and 'log2-fold change >1'. Gene set enrichment analysis was performed via the R package clusterProfiler.

QUANTIFICATION AND STATISTICAL ANALYSIS

All statistical analyses were carried out using R software (v 4.0.2). Continuous variables were compared between the two groups using T test, Welch's T test, or Wilcoxon rank-sum test. Categorical variables were compared between the two groups using Chi-Square Test. Correlations between two continuous variables were determined using Pearson or partial Spearman correlation. All statistical tests were two-sided unless otherwise stated. When analyzing immunotherapy response related gene sets, the odds ratio (OR) and p value were obtained from the meta-analysis by the R package meta. Asterisks indicate the level of statistical significance: *$p < 0.05$, **$p < 0.01$, ***$p < 0.001$, ****$p < 0.0001$, ns, not significant. For boxplots, the center line represents the median, box limits represent upper and lower quartiles, and whiskers represent 1.5 times the interquartile range. The numerical data of statistical replicates in cell lines and PDOT experiments were presented as the mean ± standard deviation (SD). Sample sizes (n) are stated in the figure or figure legend.

Cell Reports Medicine, Volume *6*

Supplemental information

Computational framework for prioritizing candidate compounds overcoming the resistance of pancancer immunotherapy

Fangyoumin Feng, Tian He, Ping Lin, Jinwu Hu, Bihan Shen, Zhixuan Tang, Jian Zhou, Jia Fan, Bo Hu, and Hong Li

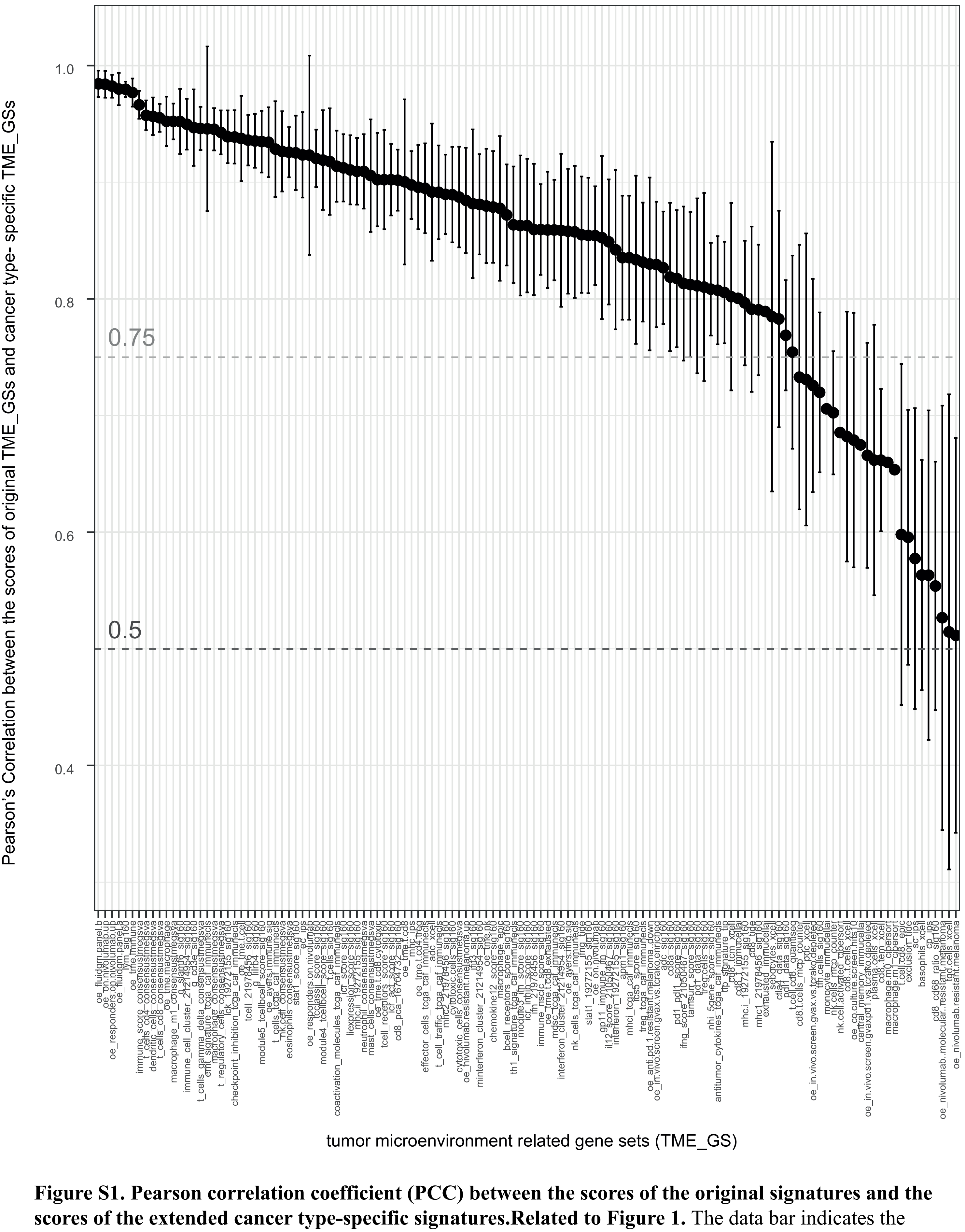

Figure S1. Pearson correlation coefficient (PCC) between the scores of the original signatures and the scores of the extended cancer type-specific signatures.Related to Figure 1. The data bar indicates the range of PCCs among multiple cancer types.

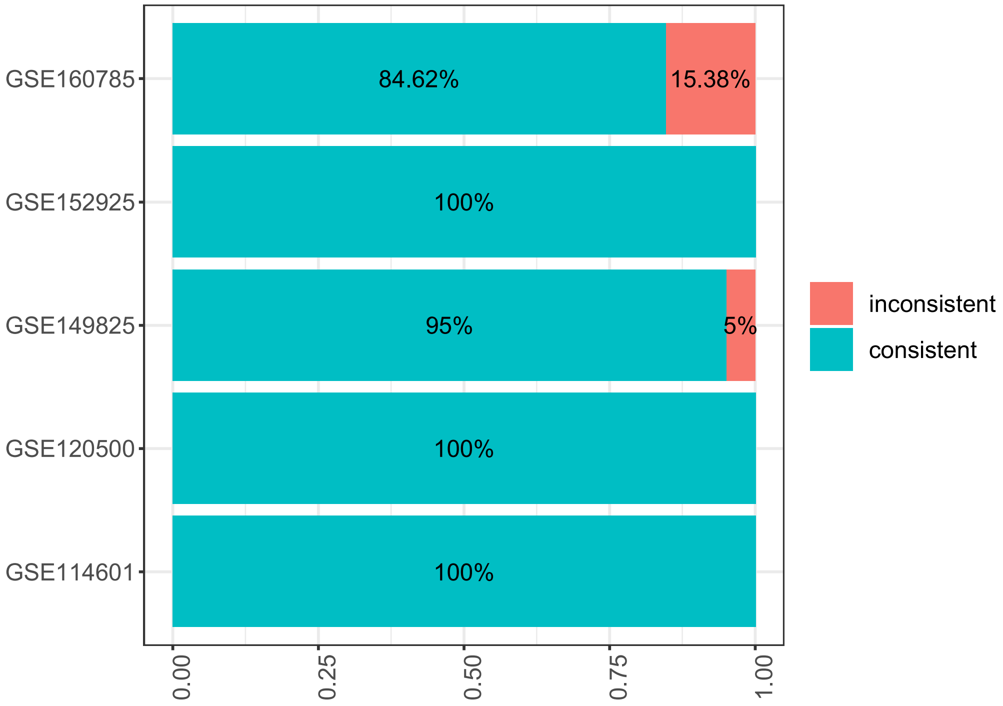

Figure S2. Consistency of drug-induced IGeS expression changes between mice and cell lines. Related to Figure 3.

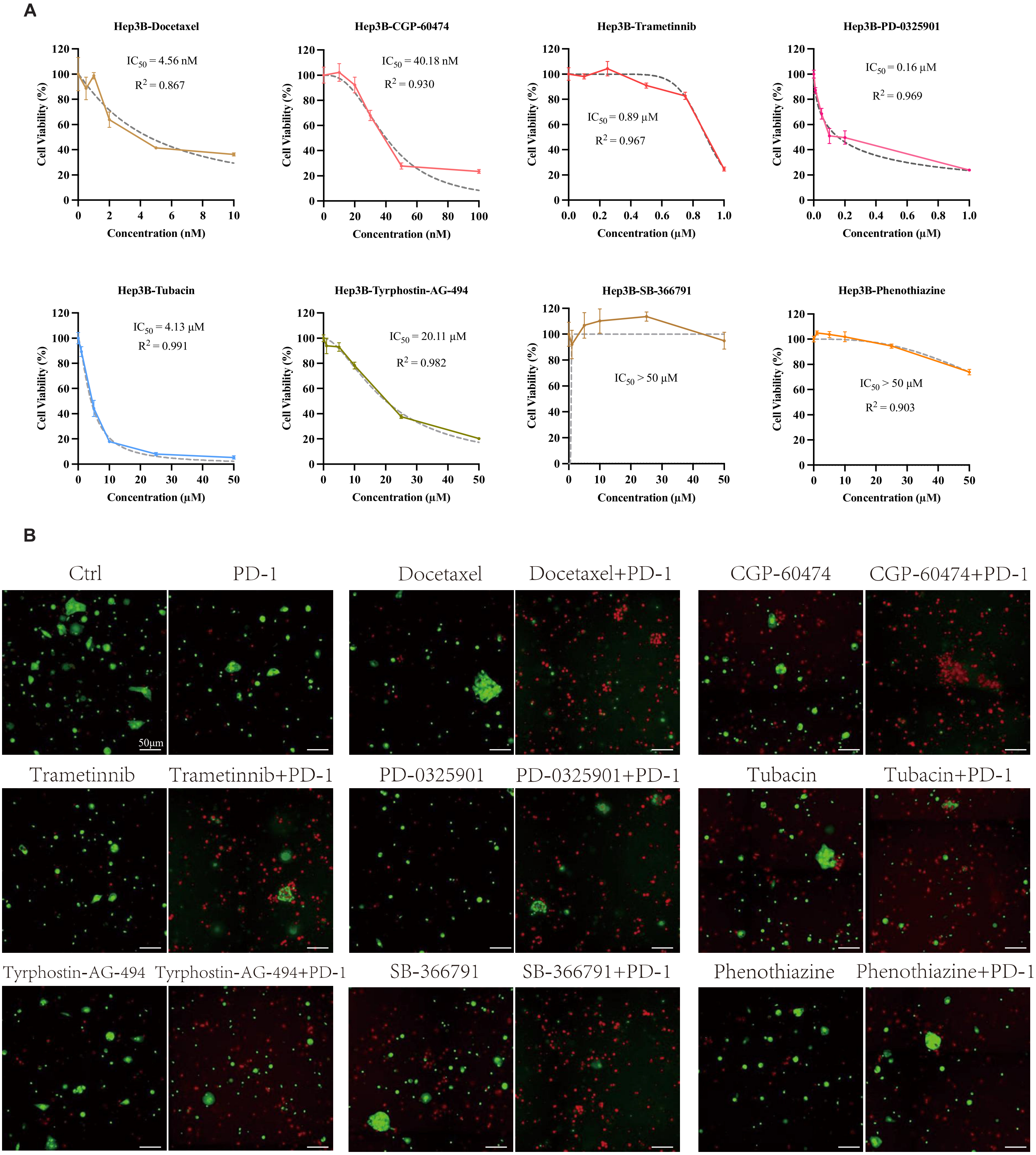

Figure S3. Drug test results in cell lines and PDOTs. Related to Figure 5. (A), The IC50 values of the selected compounds in the Hep3B cell line. **(B),** Representative pictures of PDOTs live/dead analysis via high-content screening. Live cells were stained green (AO), while dead cells were stained red (PI). Data are represented as mean ± SD. Scale bar: 50µm.

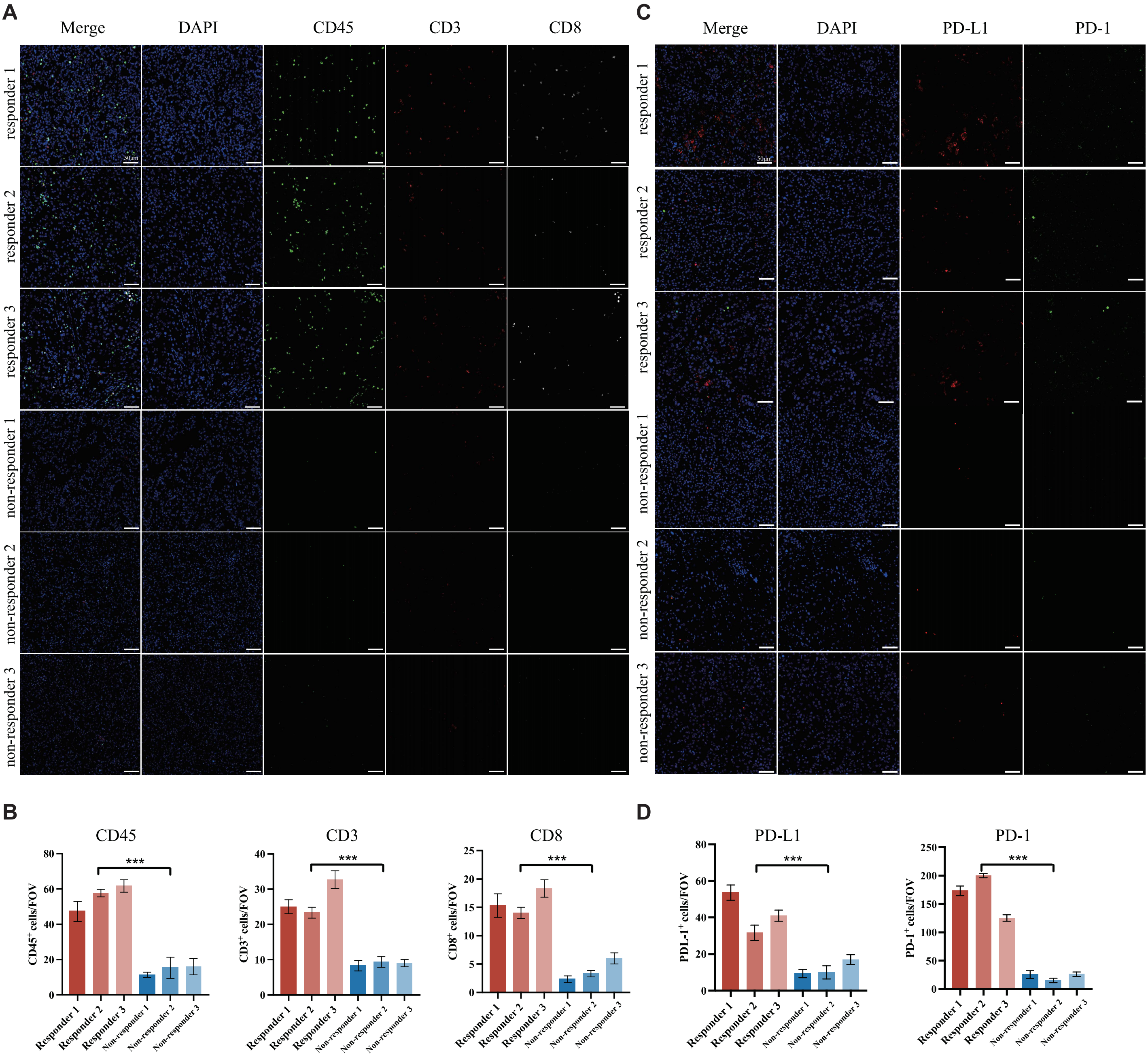

Figure S4. Comparison of immune infiltration between three anti-PD-1-resistant patients and three responding patients. Related to Figure 5. (A), Representative images of CD45, CD3 and CD8 immunofluorescence staining. **(B),** Statistical comparison of PD-1/PD-L1 expression. **(C),** Representative images of PD-1/PD-L1 immunofluorescence staining. **(D),** Statistical comparison of CD45, CD3 and CD8 expression. Scale bar: 50 µm. Data are represented as mean ± SD. Asterisks indicate the level of statistical significance: *** $p < 0.001$.

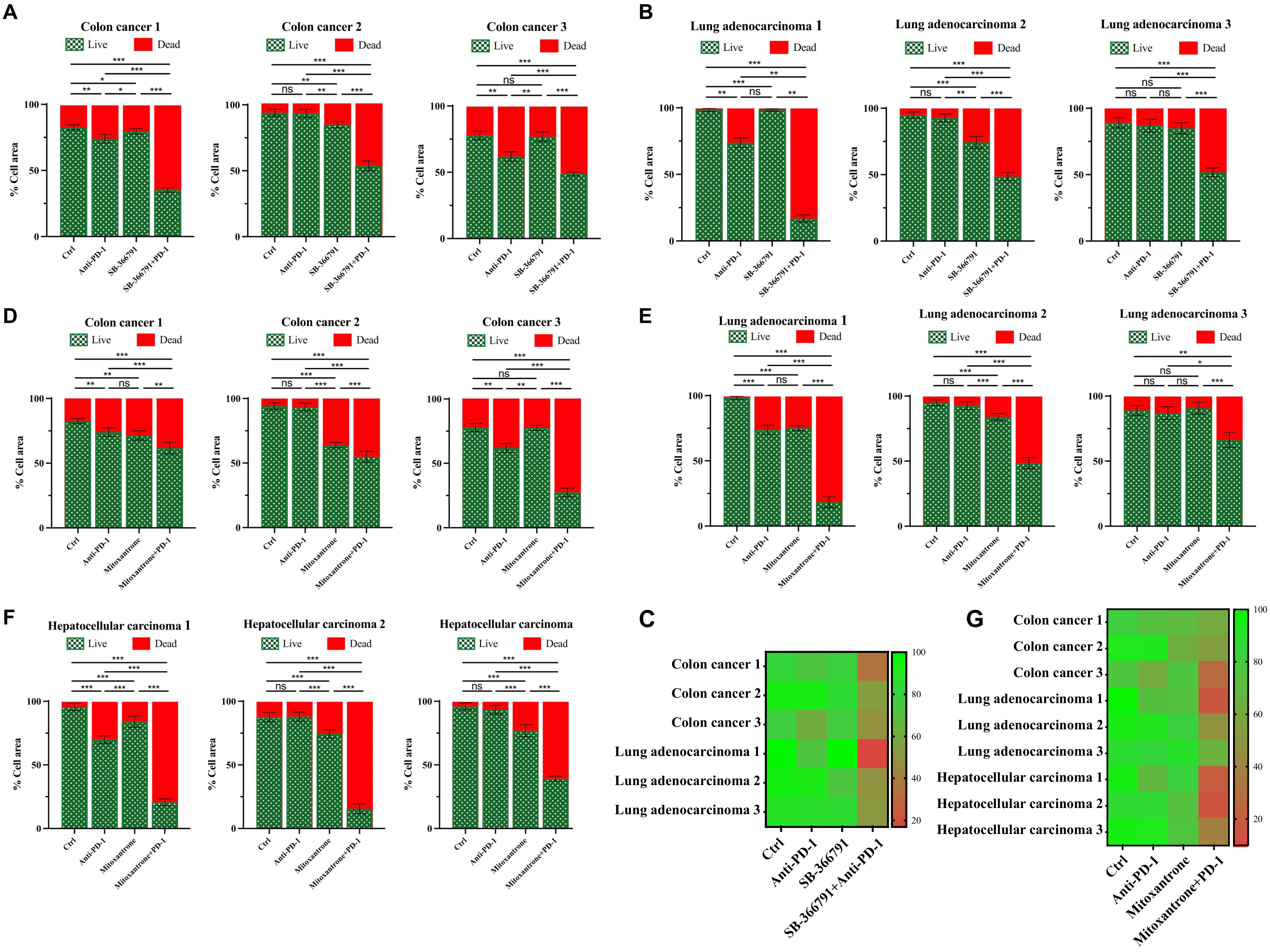

Figure S5. Validation of the effects of SB-366791 and mitoxantrone by PDOTs. Related to Figure 5. (A)-(B), PDOTs test results of SB-366791 based on colon cancer and lung adenocarcinoma samples. **(C),** Heatmap of SB-366791 PDOTs test results. **(D)–(F),** PDOTs test results of mitoxantrone in colon cancer, lung adenocarcinoma and hepatocellular carcinoma. **(G),** Heatmap of the mitoxantrone PDOTs test results. Data are represented as mean ± SD. Asterisks indicate the level of statistical significance: * $p < 0.05$, ** $p < 0.01$, *** $p < 0.001$, **** $p < 0.0001$, ns, not significant.

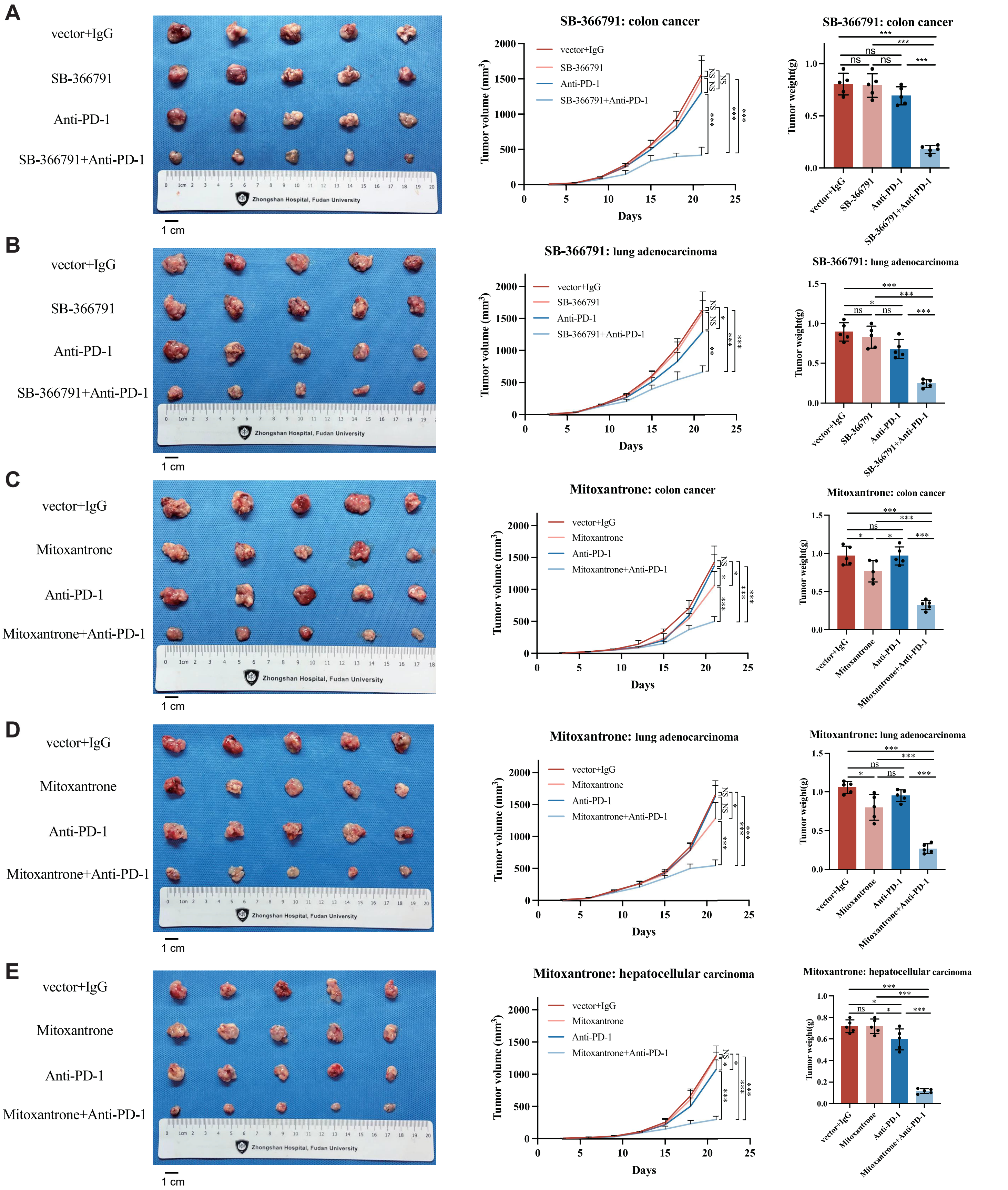

Figure S6. Validation of the effects of SB-366791 and mitoxantrone in a subcutaneous mouse model in four treatment groups: control, anti-PD-1, the candidate compound, and the combination of candidate and anti-PD-1.Related to Figure 6. (A)-(B), Results for SB-366791 in colon cancer and lung adenocarcinoma models. **(C)-(E),** Results for mitoxantrone in colon cancer, lung adenocarcinoma and hepatocellular carcinoma models. Data are represented as mean ± SD. Asterisks indicate the level of statistical significance: * $p < 0.05$, ** $p < 0.01$, *** $p < 0.001$, **** $p < 0.0001$, ns, not significant. Scale bar: 1 cm.

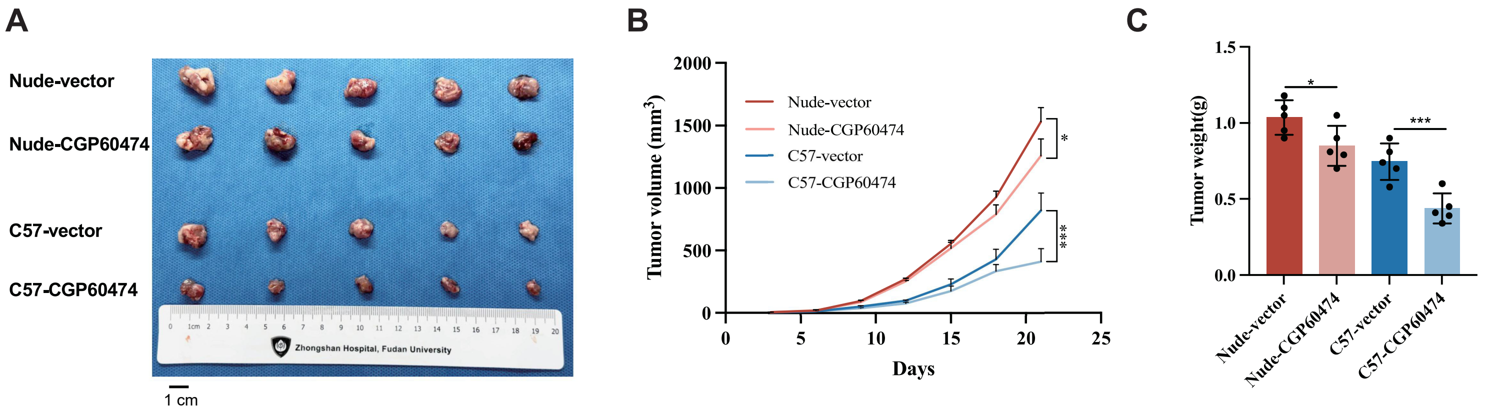

Figure S7. Representative images and quantification of subcutaneous tumors established in BALB/c nude and C57BL/6J mice. Related to Figure 6. (A), Tumour weights before and after CGP-60474 treatment. **(B),** Growth curves of the mice. **(C),** Statistical comparison of tumor weights among the different groups. To establish tumors, BALB/c nude and C57BL/6J mouse were injected subcutaneously in the right underarm with 1 × 106 Hepa1-6 cells in RPMI 1640 serum-free medium (200μL). Two weeks after injection, mice bearing subcutaneous tumors were randomized into groups. Treatments were administered CGP-60474(10 mg/kg) intraperitoneally every two days and seven times in total. Data are represented as mean ± SD. Asterisks indicate the level of statistical significance: * $p < 0.05$, ** $p < 0.01$, *** $p < 0.001$, **** $p < 0.0001$, ns, not significant. Scale bar: 1 cm.

Table S2. Manually collected immunotherapy datasets with known treatment effects and expression profiles. Related to Figures 1 and 2.

cohorts	dataset	source_PMID	cancer_type	treatment_type	responders	non-responders
discovery cohorts	D1_SKCM	26997480	SKCM (Skin Cutaneous Melanoma)	aPD1	15	12
	D2_SKCM	29033130	SKCM (Skin Cutaneous Melanoma)	aPD1	10	39
	D3_SKCM	30753825	SKCM (Skin Cutaneous Melanoma)	aPD1	19	22
	D4_SKCM	32708981	SKCM (Skin Cutaneous Melanoma)	aPD1	8	6
	D5_SKCM	27956380	SKCM (Skin Cutaneous Melanoma)	aCTLA4	8	16
	D6_KIRC	32472114	KIRC (Kidney Renal Clear Cell Carcinoma)	aPD1	39	133
	D7_KIRC	29301960	KIRC (Kidney Renal Clear Cell Carcinoma)	aPD1	8	25
	D8_LUAD	32879421	LUAD (Lung Adenocarcinoma)	aPD1	5	11
	D9_LUAD	32762727	LUAD (Lung Adenocarcinoma)	aPD1_aPDL1	8	19
	D10_STAD	30013197	STAD (Stomach Adenocarcinoma)	aPD1	12	33
	D11_BLCA	29443960	BLCA (Bladder Urothelial Carcinoma)	aPDL1	68	230
	D12_GBM	30742119	GBM (glioblastoma)	aPD1	9	7
validation cohorts	V1_SKCM	29170503	SKCM (Skin Cutaneous Melanoma)	Adoptive T-cell therapy (ACT)	10	15
	V2_SKCM	30127394	SKCM (Skin Cutaneous Melanoma)	aPD1_aCTLA4	2	9
	V3_UC	34294892	UC (Urothelial Cancer)	aPD1	16	71
	V4_SKCM	28494868	SKCM (Skin Cutaneous Melanoma)	aPD1	2	3
	V5_SKCM	31792460	SKCM (Skin Cutaneous Melanoma)	aPD1	17	24

Table S5. Mouse datasets with gene expression profiles before and after drug treatment. Related to Figure 3.

GEO	Pubmed	Drug1	Drug2	CancerType	Number of samples	SampleType
GSE149825	33589424	birinapant	anti-PD-1/CTLA-4	melanoma (primary)	12	murine melanoma mice (established B16F10 melanoma tumor mouse)
GSE152925	32723834	swainsonine	anti-PD-L1	melanoma	28	murine melanoma mice (Wildtype or Man2a1 knockout B16F10 mice)
GSE120500	30540933	olaparib		ovarian cancer	12	murine overian tumor transplanted mice (Brca1-deficient ovarian tumor bearimg mice)
GSE114601	30087114	JQ1	anti-PD-1	Non-Small-Cell Lung Cancer	8	GEMM mice (Krasþ/LSL-G12D; Trp53L/L (KP) NSCLC mouse models)
GSE160785	34031121	celecoxib	anti-PD-1	colorectal cancer	49	murine CT26 colorectal carcinoma mice (CT26 tumor Balb/c mice)

Table S8. Eight candidate compounds that may enhance the efficacy of immunotherapy for liver cancer. Related to Figure 5.

compound	MoA	FDA approved drugs	dataset	rank	LIHC_BS
CGP-60474	CDK inhibitor	no	GSE70138	3	9.021119807
PD-0325901	MEK inhibitor	no	GSE70138	4	8.586601074
trametinib	MEK inhibitor	yes	GSE70138	7	6.655274543
tyrphostin-AG-494	EGFR tyrosine kinase inhibitor	no	GSE92742	1	19.78885899
phenothiazine	Dopamine D2 receptor antagonist	yes	GSE92742	2	17.79438242
docetaxel	Tubulin inhibitor	yes	GSE92742	4	17.32441355
tubacin	HDAC inhibitor	no	GSE92742	5	17.19915011
SB-366791	TRPV1 antagonist	no	GSE92742	7	16.68389834

Note: due to less known or diifficult to obtain, compounds named only with 'BRD-' was removed from GSE92742 for selecting candidate compounds.

Table S9. Clinicopathologic features of patients for PDOTs constructions. Related to Figure 5.

# experiments of 8 compounds listed in Table S8.

hepatocellular carcinoma	Patient 1	Patient 2	Patient 3
Age,years	88	53	78
Sex	Male	Male	Female
HBsAg, COI	(-)0.274	(+)1722	(+)1677
HBV DNA, IU/mL	< 20	< 20	$1.34*10^7$
CNLC stage	Ib	Ib	Ia
BCLC stage	A	A	A
Child-Pugh	A	A	A
AFP, ng/mL	99.4	637	13.2
PIVKA-II, mAU/mL	9137	4959	378
Diameter of intrahepatic tumors, cm	6.8	6.5	4

Abbreviation: HBsAg, hepatitis B surface antigen; CNLC, China liver cancer; BCLC, Barcelona Clinic liver cancer; AFP, alpha-fetoprotein.

# experiments of mitoxantrone

hepatocellular carcinoma	Patient 1	Patient 2	Patient 3
Age,years	77	71	69
Sex	Male	Male	Male
Race	East Asian	East Asian	East Asian
HBsAg, COI	(+)9.96	(-)0.482	(+)4.36
HBV DNA, IU/mL	< 20	< 20	< 20
CNLC stage	ⅠA	ⅠA	ⅠIB
BCLC stage	A	A	B
Child-Pugh	A	A	A
AFP, ng/mL	105.3	376	59.6
PIVKA-II, mAU/mL	222	460	33
Diameter of intrahepatic tumors, cm	64.2	4.5	5

# experiments of SB-366791 and mitoxantrone

colon cancer	Patient 1	Patient 2	Patient 3
Age,years	67	80	57
Sex	Male	Male	Female
Race	East Asian	East Asian	East Asian
Tumor sidedness	Right	Left	Right
Tumor stage	IIIA	IIIB	IIIA

# experiments of SB-366791 and mitoxantrone

lung adenocarcinoma	Patient 1	Patient 2	Patient 3
Age,years	61	59	49
Sex	Male	Male	Female

Race	East Asian	East Asian	East Asian
Tumor stage	IIB	IIA	IIA
